# Single-Cell and Spatial Transcriptomics Across Populations With Perianal Fistulizing Crohn’s Disease Shows Proinflammatory CD4^+^ Macrophage Involvement in Disease Progression

**DOI:** 10.1016/j.jcmgh.2026.101862

**Published:** 2026-08-12

**Authors:** Sushma C. Maddipatla, Sachith Munasinghe, Anne Dodd, Christopher Tastad, Rachel Moss, Hong Yin, Shanta Murthy, Abhishek Dash, Vasantha L. Kolachala, David J. Cutler, Judy Cho, Subra Kugathasan, Jason D. Matthews

**Affiliations:** 1 Division of Gastroenterology, Hepatology, and Nutrition, Department of Pediatrics, Emory University School of Medicine, Atlanta, Georgia; 2 Department of Pathology, Molecular, and Cell-Based Medicine, Icahn School of Medicine at Mount Sinai, New York, New York; 3 Department of Pathology, Children’s Healthcare of Atlanta, Atlanta, Georgia; 4 Department of Human Genetics, Emory University School of Medicine, Atlanta, Georgia; 5 Department of Pediatric Gastroenterology, Children’s Healthcare of Atlanta and Pediatric Research Institute, Atlanta, Georgia

**Keywords:** Ancestry, CD4, Crohn's Disease, Macrophages, Myleoid, Perianal Fistula, Single-Cell, Xenium

## Abstract

**Background & Aims:**

Perianal fistulizing Crohn’s disease is a severe morbidity with rectal involvement and a higher prevalence in African Americans compared with European Americans. In this study, we sought to define the distinguishing cellular and molecular features between inflamed rectal Crohn’s disease with and without perianal fistula across populations that give new insight into disease severity or progression.

**Methods:**

Rectal mucosal biopsies were obtained for single-cell sequencing from 50 individuals (262k cells); 18 with Crohn’s disease of the rectum with fistula, 25 with Crohn’s disease of the rectum without fistula and any other perianal complications, and 7 from individuals with no past or present mucosal disease (controls). Spatial transcriptomics were conducted and analyzed by Sopa and Squidpy, whereas immunofluorescence was processed with QuPath. Patient-derived rectal organoids were used to test inflammatory signaling.

**Results:**

A major distinction between inflamed Crohn’s disease of the rectum with and without fistula was the decreased phagocytic signature, antigen processing profile, and unique morphologic structures of proinflammatory CD4^+^CD68^+^ macrophages. In fistulizing Crohn’s disease, these macrophages formed large aggregates in the lamina propria near epithelial crypts and appeared to disrupt cellular networks by engaging in signaling with other cell subtypes. At the population level, the most significant differences in cellular pathways were detected between African Americans and European Americans with inflamed Crohn’s disease of the rectum and fistula, and between patients with and without fistulizing disease among African Americans.

**Conclusions:**

During inflamed Crohn’s disease of the rectum with fistula, CD4^+^ macrophages alter cellular networks in the lamina propria while forming large cell aggregates, likely mediating complex interactions with T cells and possibly microbial antigens that are involved in the more severe pathologic conditions associated with fistula tract formation and progression.

**Summary**

This study shows that hyperactive immune responses in T cells and changes in epithelial cell function are associated with macrophage-mediated antigen processing in perianal fistulizing Crohn’s disease.

**What You Need to Know**

**Background**

Perianal fistulizing Crohn’s disease is anatomically characterized by having rectal involvement. Our results suggest that T cells that are associated with changes in crypt epithelial cell-specific MHC class II activity and CD4^+^ macrophage behavior in the lamina propria.

**Impact**

These findings implicate T-cell signaling and macrophage antigen processing as potential clinical targets to be explored in future studies. Mitigating these changes in cellular function could protect the epithelial cells and restore their normal function, a critical step in mucosal healing.

**Future Directions**

These findings should motivate deeper investigations into factors stimulating interferon pathways in the rectal mucosa and the different aspects of epithelial cell–macrophage Dcrosstalk during Crohn’s disease.

Crohn’s disease (CD) is a chronic inflammatory condition of the gastrointestinal tract, commonly affecting the terminal ileum and proximal colon.^1^, ^2^, ^3^, ^4^, ^5^, ^6^, ^7^ CD affecting the distal colon, particularly the rectum, is associated with severe and aggressive complications such as perianal fistulizing CD. The presence and anatomic location of the deep fistula tracts with external opening into the perineal area distinguish this extreme phenotype,^5^^,^^6^ setting it apart from the stricturing luminal disease that is often found in severely inflamed (IF) small intestinal CD.^2^

Perianal fistulizing CD is highly prevalent in African American (AA) populations, with worse outcomes compared with those with European American (EA) ancestry.^8^ Although CD has many known genetic associations,^9^^,^^10^ there have not been any associations detected for the perianal fistulizing CD phenotype, suggesting that cellular and/or environmental factors are likely to play a more prominent role in promoting perianal complications.^11^ Our previous studies support epithelial cell dysfunction playing a role in perianal fistulizing Crohn’s disease, including epithelial cell pathways associated with metabolism, proliferation, and goblet cell function.^12^^,^^13^ These are consistent with previous reports that have pointed to changes in rectal epithelial cells that are involved with forming the fistula tract through mechanisms like epithelial-to-mesenchymal transitioning.^14^, ^15^, ^16^, ^17^, ^18^, ^19^, ^20^ Recently, CD14^+^ myeloid cells and their crosstalk with inflammatory fibroblasts were also functionally implicated in perianal fistulizing CD, confirming immune–stromal involvement in the pathogenesis of this phenotype.^21^ Thus, further investigations into the cellular mechanisms driving this phenotype in the rectum are warranted.

To that end, in this study, we compared CD of the rectum with and without perianal fistula along with non-inflammatory bowel disease (IBD) controls across ancestral populations at the single-cell level to determine the distinguishing molecular and cellular features among the phenotypes while gaining insight into the interplay of the epithelial, immune, and stromal compartments and their potential impact on the progression of CD of the rectum towards fistula development. We identified transcriptional and morphologic changes pointing to differences in CD4^+^/CD68^+^ macrophages, changes in the activity of the stromal compartments, and disruption of crosstalk between epithelial cells, along with clear distinctions in the immune pathways and cytokine profiles between CD of the rectum with and without fistulizing disease. Some of these differences reside at the population level, whereas others showed differences in the cellular networks connecting mucosal neighborhoods. Our results indicate that specialized proinflammatory macrophages play a role in the pathology of fistula development in CD of the rectum that is involved in a type of antigen processing not observed in CD of the rectum without fistula.

## Results

### Integrative Single-Cell Portal for Crohn’s Disease of the Rectum and Perianal Complications

Subjects with CD of the rectum with and without perianal fistulizing disease were recruited for the study, and their rectal mucosal biopsies were analyzed by single-cell RNA sequencing (scRNA-seq) (Figure 1*A–C*). Roughly one-half of the patient cohort were of EA ancestry, with the remaining having primarily (∼45%) AA and ∼7% Asian ancestry (Figure 1*B*). About 40% of the cohort had an inflammatory phenotype (10 CD of the rectum without fistula and 7 CD of the rectum with fistula) with visibly active disease at the time of endoscopic biopsy (Figure 1*B* and Figure 2). After quality control (QC) filtering, the resulting 262,849 cells, mapping to 27 cell types from 50 of the recruited individuals, were available for downstream analysis (Figure 1*C* and Figure 3).

**Figure 1:**
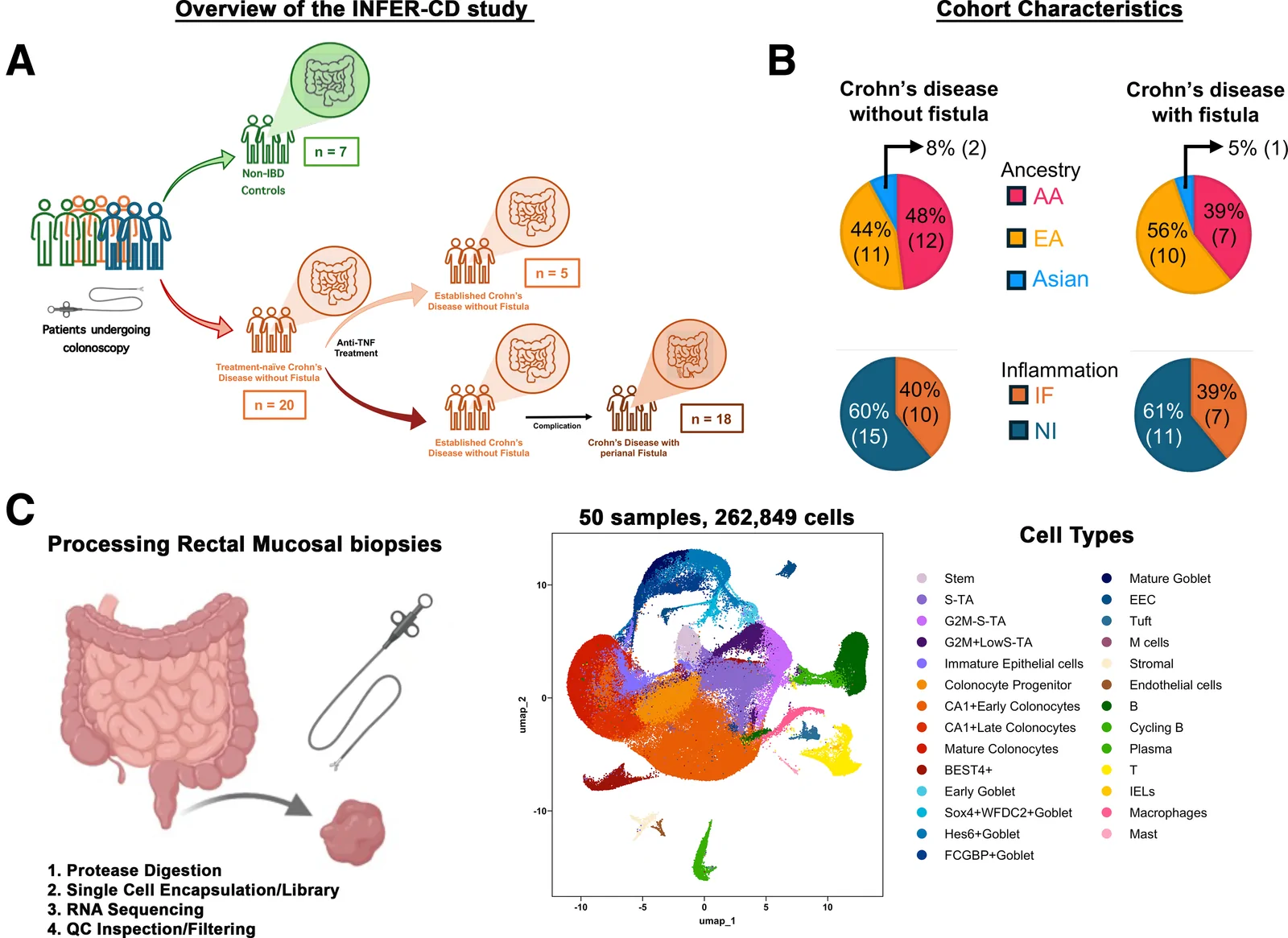
**An overview of the INFER-CD (Inflammatory Networks and Fistula-associated Epithelial Remodeling in Crohn’s Disease) study.** (*A*) Study design illustrating CD phenotypes and the number of patients representing each phenotype. (*B*) Patient clinical characteristics included in the study among patients with CD with and without perianal fistula, represented as pie charts. (*C*) Workflow illustrating rectal biopsy processing, single-cell dissociation, and UMAP visualization of integrated scRNA-seq data with 27 annotated cell types after QC (262,849 cells).

**Figure 2:**
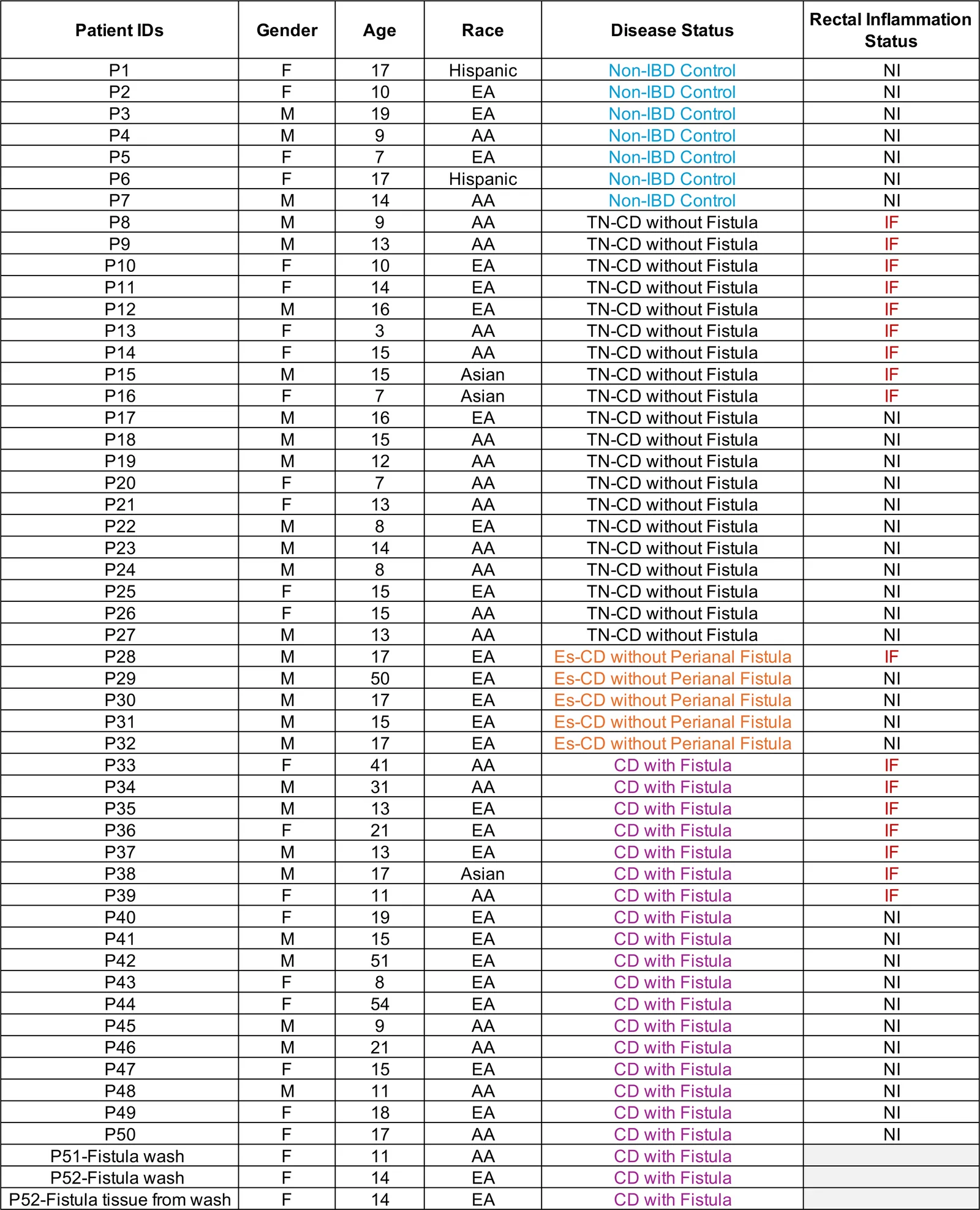
**Patient demographics and clinical characteristics.** Overview of patient samples included in this study, detailing gender, age at diagnosis, ancestry, and inflammation status of rectal mucosal biopsy specimens.

**Figure 3:**
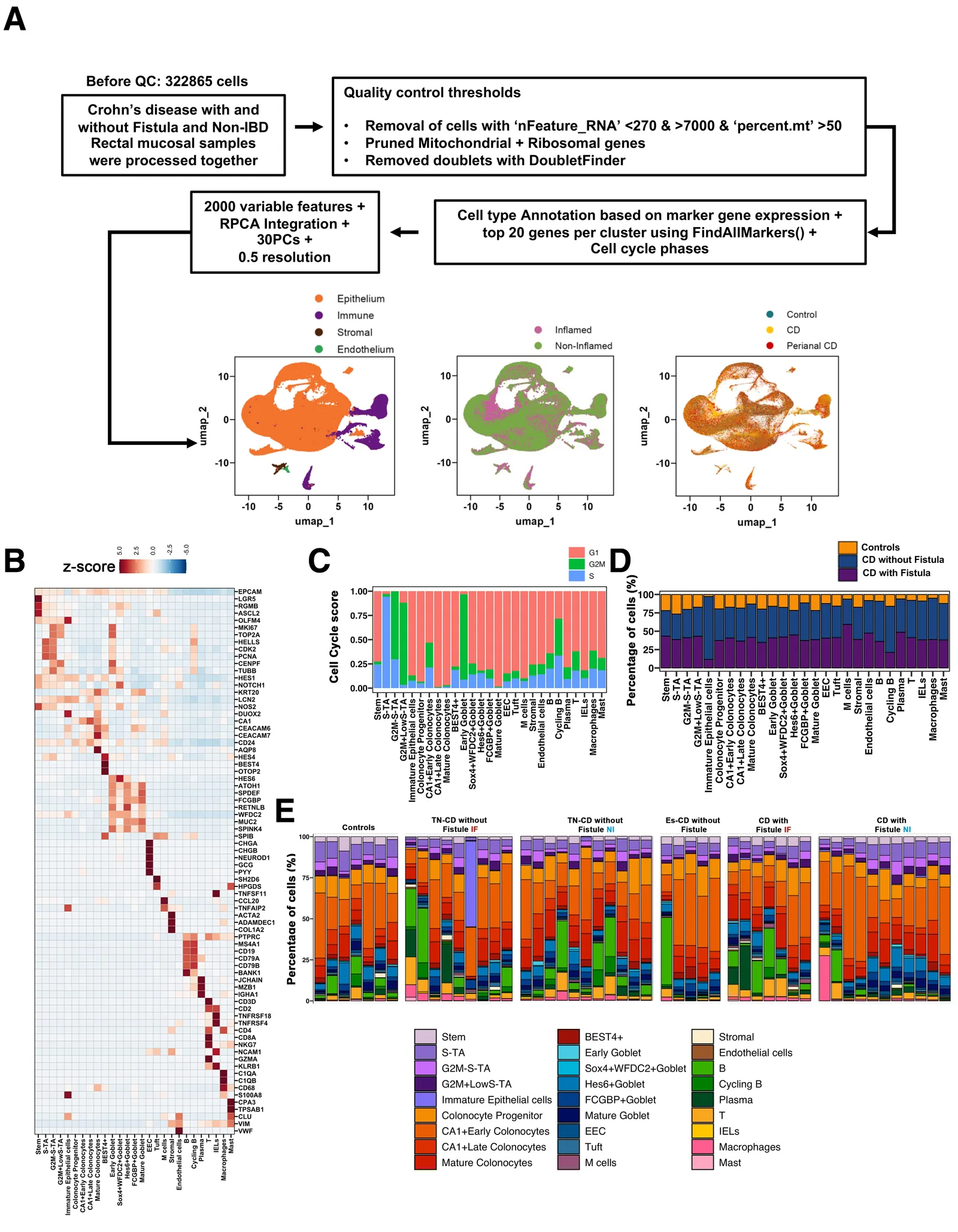
**QC and annotation of rectal mucosal single-cell data.** (*A*) Schematic illustrating preprocessing steps and QC thresholds applied to rectal single-cell data. (*B*) Heatmap showing relative expression (z-score normalized) of selected marker genes used for cell-type annotation. (*C*) Stacked bar plot depicting cell-cycle phase distribution across rectal mucosal cell types. (*D*) Proportion of each rectal cell type derived from non-IBD controls and patients with CD with and without fistula. (*E*) Distribution of cell-type fractions across individual samples.

### Mucosal Pathways, Cellular Composition, and Gene Expression in Inflamed Crohn’s Disease of the Rectum With and Without Fistula

We assessed single-cell profiles across the phenotypic groups (Figures 4 and 5) and evaluated the effects that implicated molecules had on rectal epithelial cells using organoids. Subsequent single-cell analysis across the CD of the rectum phenotypes showed cellular proportions to be largely similar, except for IF CD of the rectum with and without fistula showing increased proportions of B and T cells, and an immature epithelial subtype enriched in IF CD of the rectum without fistula (Figure 4*A*; Figure 3). These enrichments in adaptive cell proportions were also reflected in the inflammatory gene scoring profiles of the IF disease samples that showed higher scores (Figure 4*B*; Figure 5). The presence of mucosal inflammation was also the factor explaining the highest degree of variance among the cohort (Figure 4*C* and Figure 5). The crypt–axis scoring for IF CD of the rectum with and without fistula showed a shift towards crypt top–differentiated epithelial cells compared with non-IBD controls (Figure 4*D*). Indeed, cell percentages revealed a decrease in the lower transit amplifying (S-TA) compartment of the crypt in CD of the rectum with and without fistula (Figure 4*E*).

**Figure 4:**
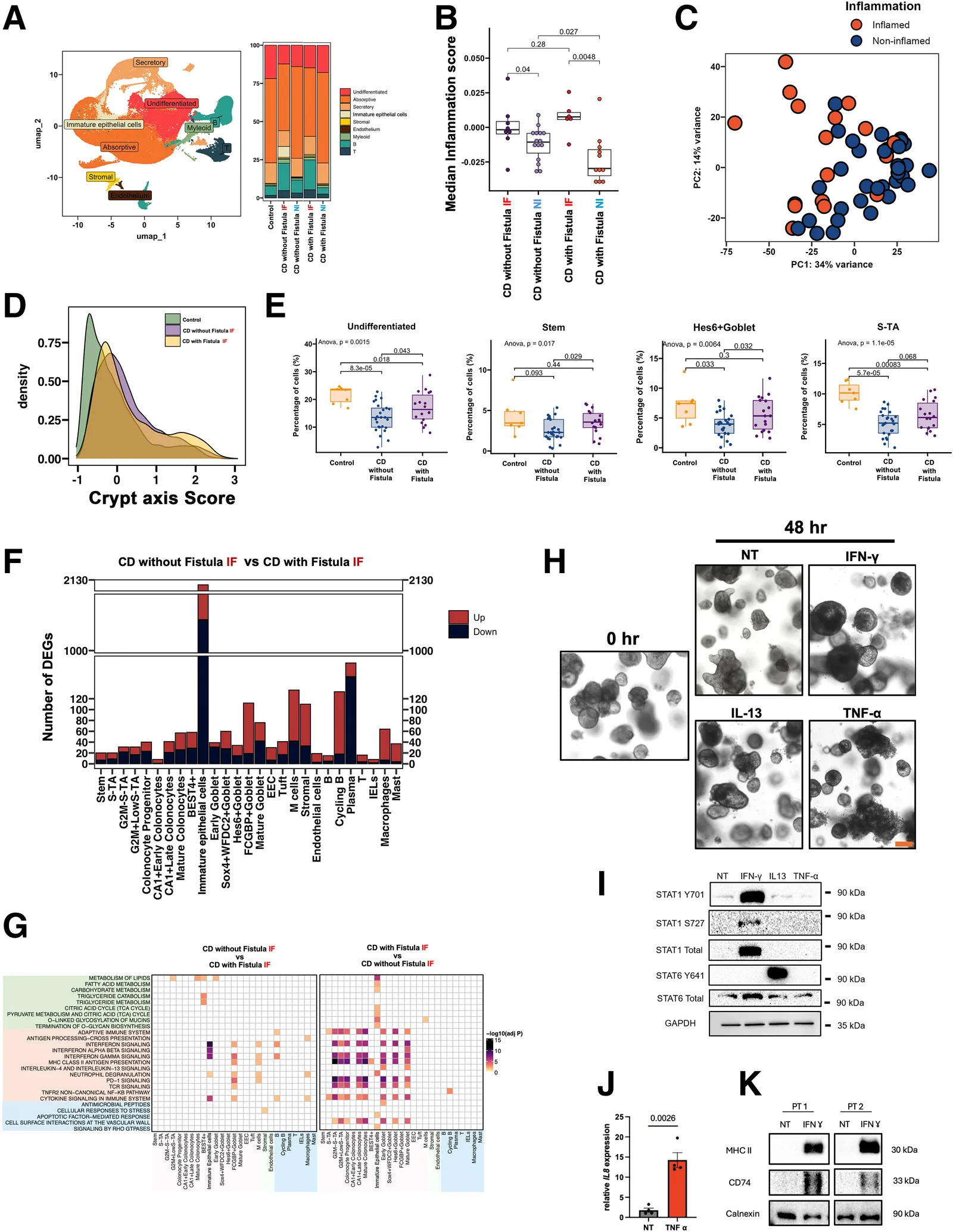
**Cellular proportions and pathways in perianal CD.** (*A*) UMAP visualization of major cell clusters in integrated scRNA-seq data and the corresponding bar plot showing the relative cell proportions across each disease phenotype. (*B*) Box and whisker plot showing significantly higher median transcript-level inflammation scores in IF CD of the rectum without perianal complications and IF CD of the rectum with perianal complications, compared with their respective NI counterparts (*P* < .05 [analysis of variance]). (*C*) PCA showing PC1 and PC2 variance color-coded by the rectal mucosal inflammatory phenotype. (*D*) Crypt axis score showing a right-ward shift in IF CD of the rectum with and without fistula relative to non-IBD controls, indicating a lower abundance of nondifferentiated cells in both CD groups compared with non-IBD controls. (*E*) Box and whisker plots showing the abundance of annotated undifferentiated cells, stem cells, HES6^+^ goblet cells, and secretory TA (S-TA) cells in non-IBD controls and patients with CD with and without fistula in integrated RNA-seq data (*P* < .05 [analysis of variance]). (*F*) Number of differentially expressed genes between CD without fistula and CD with fistula in IF rectum. (*G*) Heatmap showing the results of pathway analysis, highlighting selected significantly upregulated pathways across different cell clusters in IF CD of the rectum without perianal complications and CD of the rectum with perianal complications. (*H*) Representative images of organoids when stimulated with IFN-γ, IL13, and TNF-α after 48 hours, or untreated. (*I*) Western blot analysis of rectal organoids stimulated with IFN-γ (1 ng/mL), IL13 (100 ng/mL), and TNF-α (25 ng/mL) for 48 hours. (*J*) Bar graph highlighting the significantly higher *IL8* relative expression in rectal organoids treated with TNF-α (25 ng/mL) for 48 hours (simple *t* test). (*K*) Western blot analysis of MHCII expression in (n = 3 biological replicates) rectal organoids after 48-hour IFN-γ stimulation.

**Figure 5:**
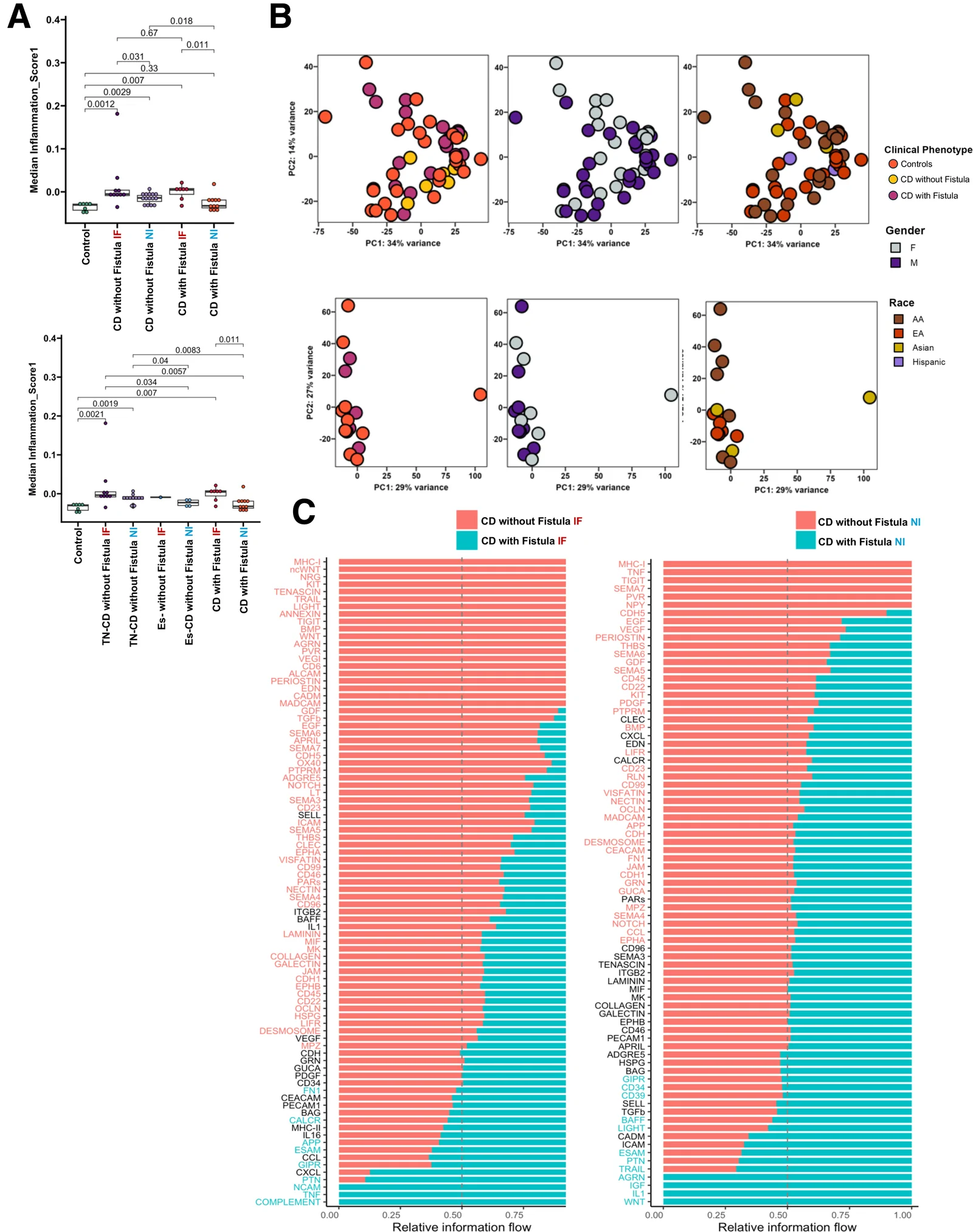
**Distinctions between CD phenotypes with or without fistula and non-IBD controls.** (*A*) Box plot showing the median inflammation scoring across broad and narrow phenotypes. (*B*) Pseudobulk PCA plot illustrating the PC1 and PC2 variance, colored by clinical phenotype, gender, and ancestry. (*C*) Stacked bar plot showing enrichment of cell–cell communication signaling pathways, with each pathway colored according to the phenotype in which it is significantly enriched.

Given that the origins of perianal fistulizing CD likely reside within the microenvironments caused by an IF rectum, we were most interested in the cellular differences between the IF CD of the rectum with and without fistula. Among those individuals, an epithelial precursor subtype with an immature epithelial cell–like transcriptome had an unusually large difference in the number of differentially expressed genes (Figure 4*F*), likely due to the expression of multiple transitionary genes taking place within the TA compartment of the crypts. Nonetheless large changes in gene expression levels for FCGBP^+^ goblet, microfold epithelial, stromal, cycling B, plasma, and mast cells, along with macrophages, were observed (Figure 4*F*). The most remarkable differences in the pathway analysis between IF CD of the rectum with and without fistula were the upregulation of inflammatory pathways in epithelial subtypes in fistulizing disease, including those related to the vasculature wall interactions, programmed cell death protein 1 (PD-1), T-cell receptor (TCR), adaptive immune system, interferon (IFN)-γ/cytokine signaling, interleukin (IL)13, and MHC class II (MHCII) antigen presentation (Figure 4G). Additionally, cell–cell communication pathways involving MHC class I, metabolism of lipids, noncanonical WNT signaling, and bone morphogenic protein were downregulated with elevated complement and tumor necrosis factor (TNF) pathways in IF CD of the rectum with fistula. (Figure 5).

Considering these findings, we independently tested the effect of each mediator, IFN-γ, TNF-α, or IL13, directly on rectal organoids (Figure 4*H* and *I*). We found that IFN-γ–treated organoids had larger, darker spheroids, than did untreated or IL13-treated organoids, and TNF-α–exposed organoids were dark and more necrosed (Figure 4*H*). In terms of signaling, we found that IFN-γ stimulated the signal transducer and activator of transcription (STAT)1 pathway, and IL13 stimulated the STAT6 pathway, with no detectable response in these pathways to TNF-α (Figure 1). However, rectal organoids significantly increased their expression of *IL8* in response to TNF-α under these same experimental conditions (Figure 4*J*). Because STAT1 is known mediator of MHCII expression, we confirmed this effect by Western blotting on additional rectal organoids derived by endoscopic biopsy from other individuals. Figure 4*K* shows the expression of MHCII and CD74 after IFN-γ exposure. T cells were the predominant source of IFN-γ in IF CD of the rectum with and without fistula, with higher expression in fistulizing disease. Interestingly, some IFN-γ expression was uniquely originating from epithelial M and stem cells in IF CD of the rectum with fistula, or TA and immature epithelial cells in IF CD of the rectum without fistula (Figure 6).

**Figure 6:**
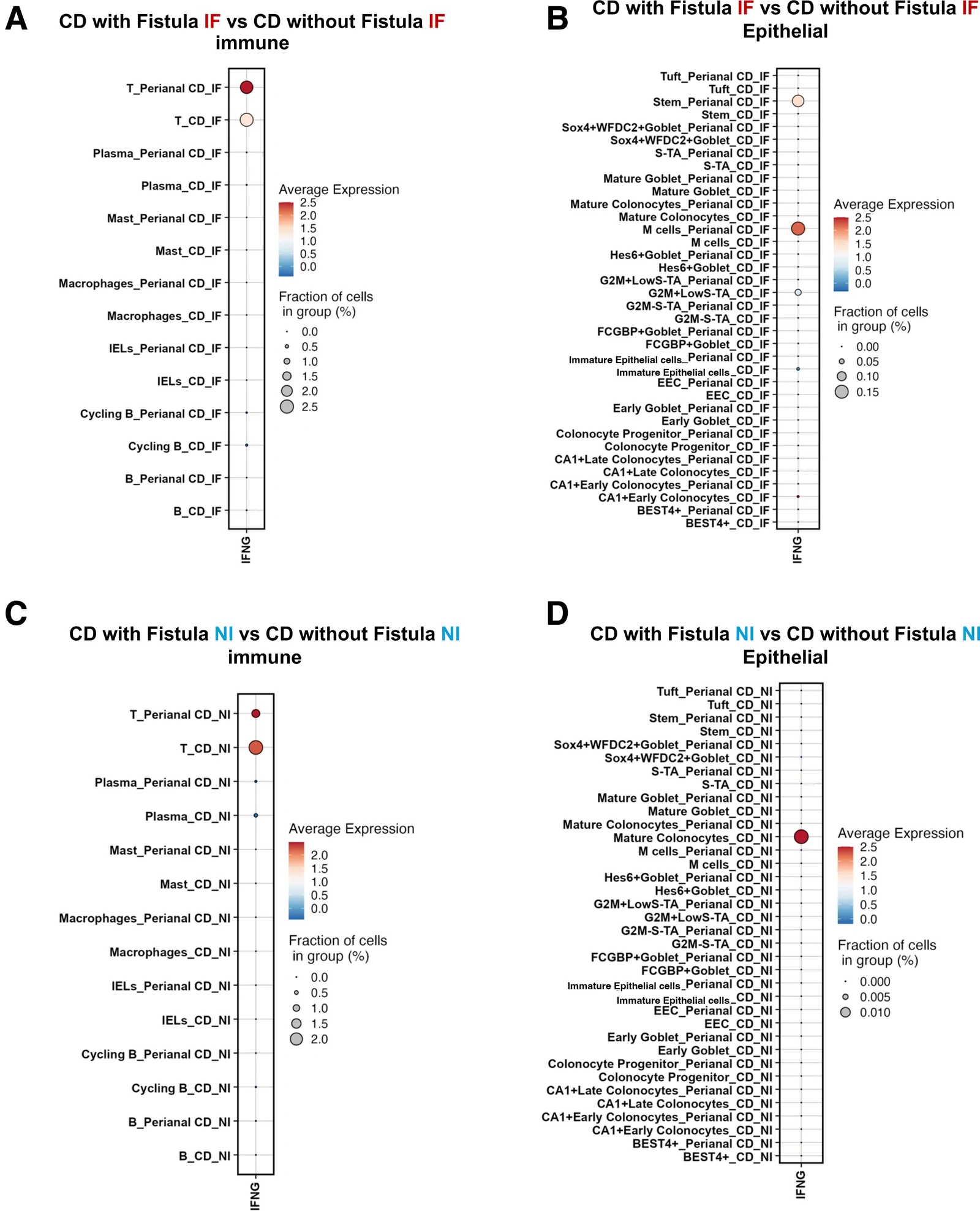
**Cellular sources of IFNG in rectal mucosal tissue.** (*A–D*) Dot plot showing relative IFNG expression across immune and epithelial compartments in IF and NI CD samples with or without fistula.

### Cellular Pathways Associated With Inflamed Crohn’s Disease of the Rectum With and Without Fistula Across Ancestral Populations

Next, we examined the differences across the ancestry groups in our cohort with active disease marked by inflammation observed at the time of endoscopy (Figure 7; Figure 8). Pathway analysis generated from scRNA-seq data of these samples showed that the most striking differences were observed in the AA individuals with IF CD of the rectum with fistula (Figure 7*A* and *B*). First, to detect ancestry-dependent variabilities, we investigated the differences between AA patients with IF CD of the rectum with fistula and EA patients with IF CD of the rectum with fistula. We observed large differences between these 2 groups involving inflammatory pathways in the epithelial compartments. Notably, pathways involving cytokine signaling, IFN signaling, and MHCII antigen presentation were significantly enriched in AA patients with IF CD of the rectum with fistula. These differences were noted in both immature and mature cells, suggesting a remodeling along the crypt axis with more prominent inflammatory signaling in the epithelial compartment of AA patients. Along with this, antimicrobial peptide–associated pathways were enriched in AA patients with IF CD of the rectum with fistula compared with EA patients. The stromal compartment in EA patients with IF CD of the rectum with fistula had increased cytokine and IL12 and IL17 signaling compared with AA patients with IF CD of the rectum and fistula (Figure 7*A*).

**Figure 7:**
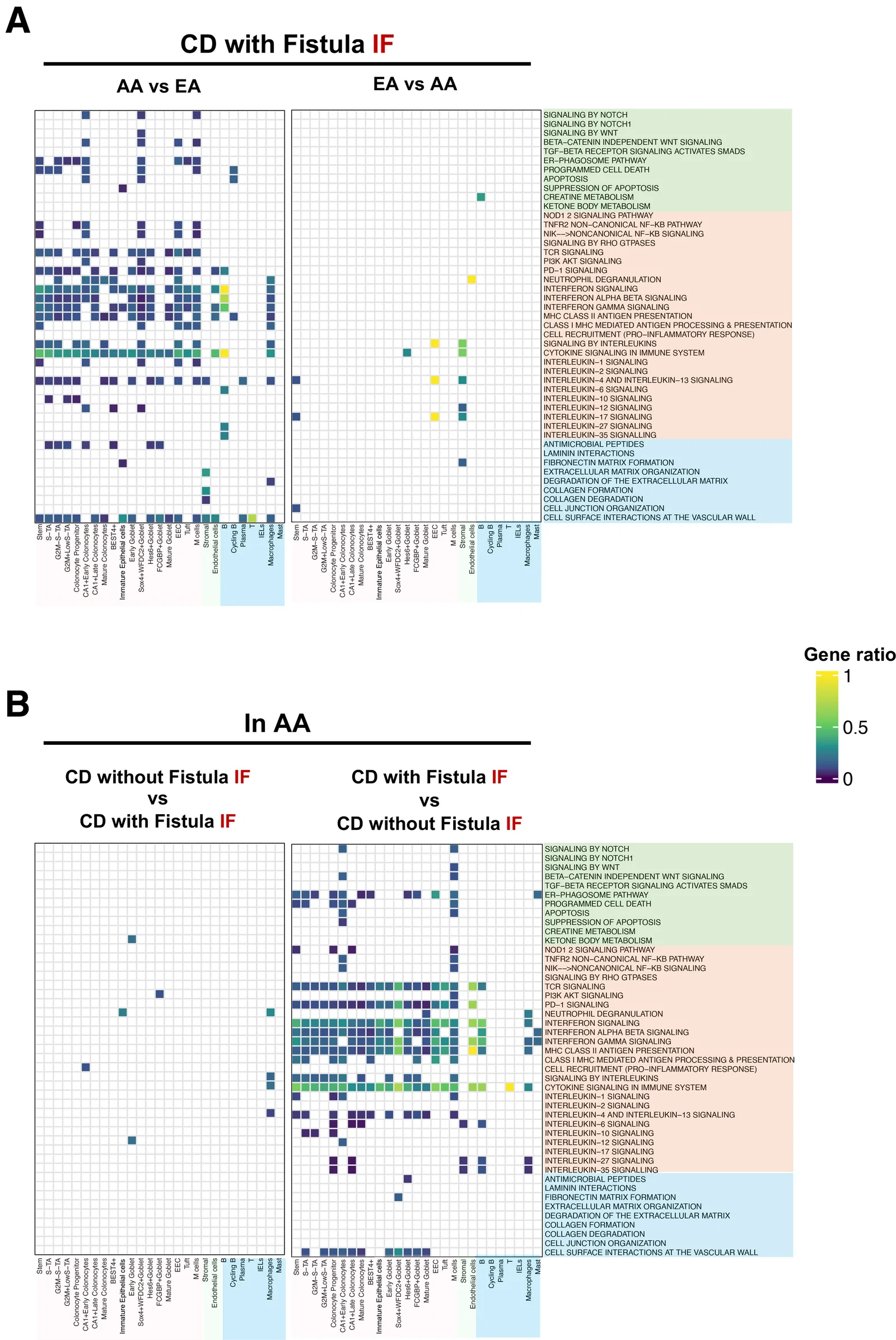
**Pathway analysis with single cells from AA and EA with inflamed CD of the rectum with and without fistulizing phenotype.** (*A* and *B*) Bioanalytical analysis of pathways changing across multiple phenotypic and population-specific variables during inflamed disease. Variables compared in the subanalysis are listed above the heat map.

**Figure 8:**
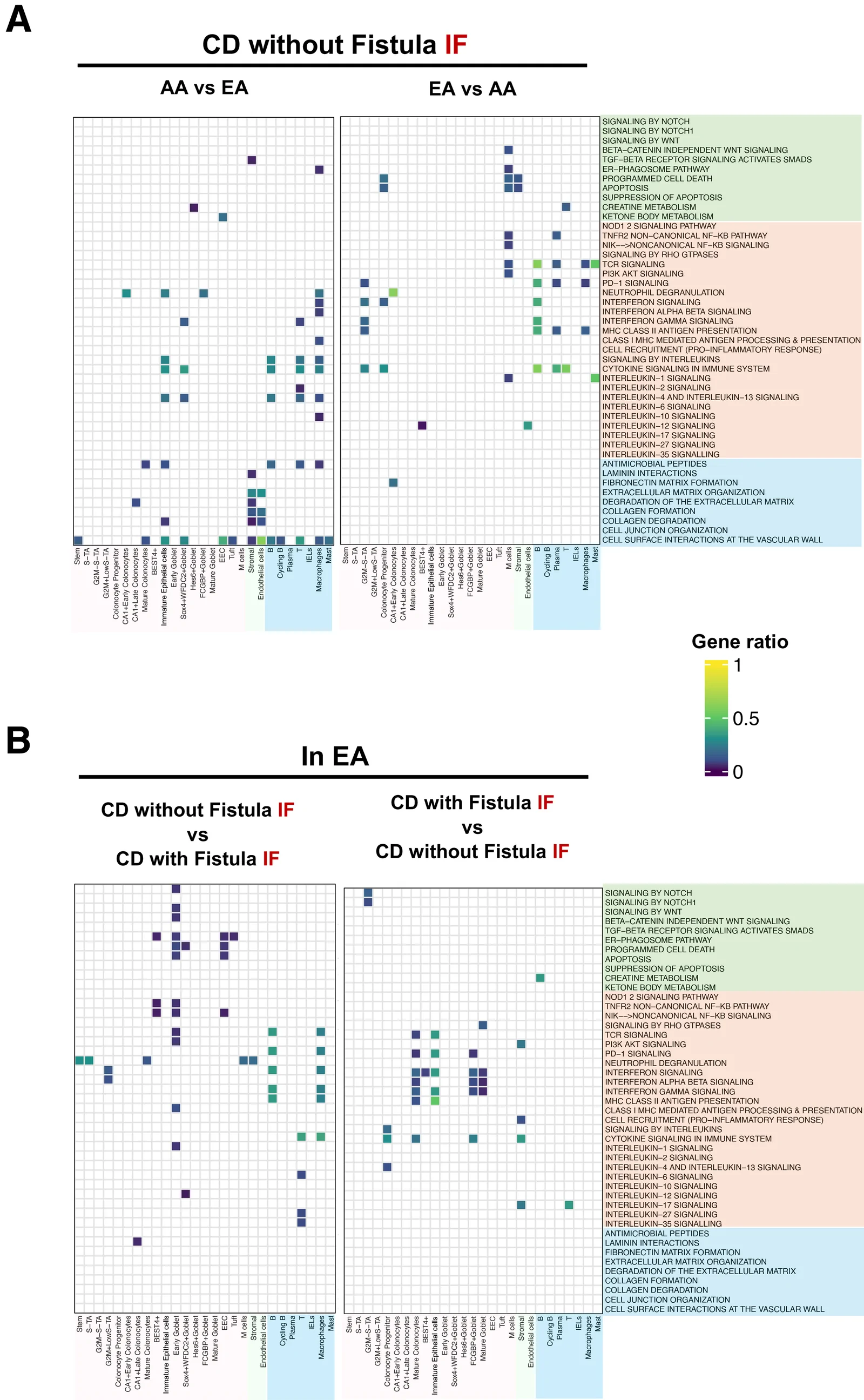
**Pathways enriched across the AA and EA ancestries in the IF CD of the rectum without fistula group.** (*A*) Heatmap showing the selected significantly enriched pathways across ancestry comparisons in IF CD of the rectum without fistula. (*B*) Pathway enrichment within the EA population and comparison between IF CD of the rectum with and without fistula.

Next, to investigate the AA cohort, we examined the differences between IF CD of the rectum with and without fistula (Figure 7*B*). Like our findings above, patients with fistula had signaling pathways that were distinct from their nonfistulizing counterparts. Other than the inflammatory pathways described above, unique pathways such as TCR and PD-1 signaling were enriched in the epithelium of AA patients with IF CD of the rectum with fistula. Another notable difference was the upregulated immune pathways involving IL27, IL36, and IL6 signaling in the stromal compartment of AA patients with CD with fistula along with other inflammatory pathways (Figure 7*B*).

When comparing AA patients with EA patients, vascular wall interactions in the endothelial cell compartment were upregulated in the AA group with CD of the rectum without fistula when compared with EA patients with a similar phenotype (Figure 8*A*). Comparing CD of the rectum with and without fistula within the EA group showed increased MHCII antigen presentation from mature colonocytes and lower-crypt TA epithelial cells in IF CD of the rectum with fistula (Figure 8*B*).

### Cellular Networks in Inflamed Crohn’s Disease of the Rectum With Fistula

Next, we examined the effects of inflammation on receptor–ligand signaling (Figures 9 and 10). We quantified the total number of inferred cell–cell interactions broadly among the phenotypes, revealing no statistical differences but a notable decrease in the number of cellular interactions for individuals with IF CD of the rectum with fistula (Figure 9*A*). We further examined the direction and magnitude of signals between cell types and compartments (Figure 10) within IF CD of the rectum with and without fistula. This analysis suggests that the number of signals between cell types in fistulizing disease were diminished (Figure 9*B*).

**Figure 9:**
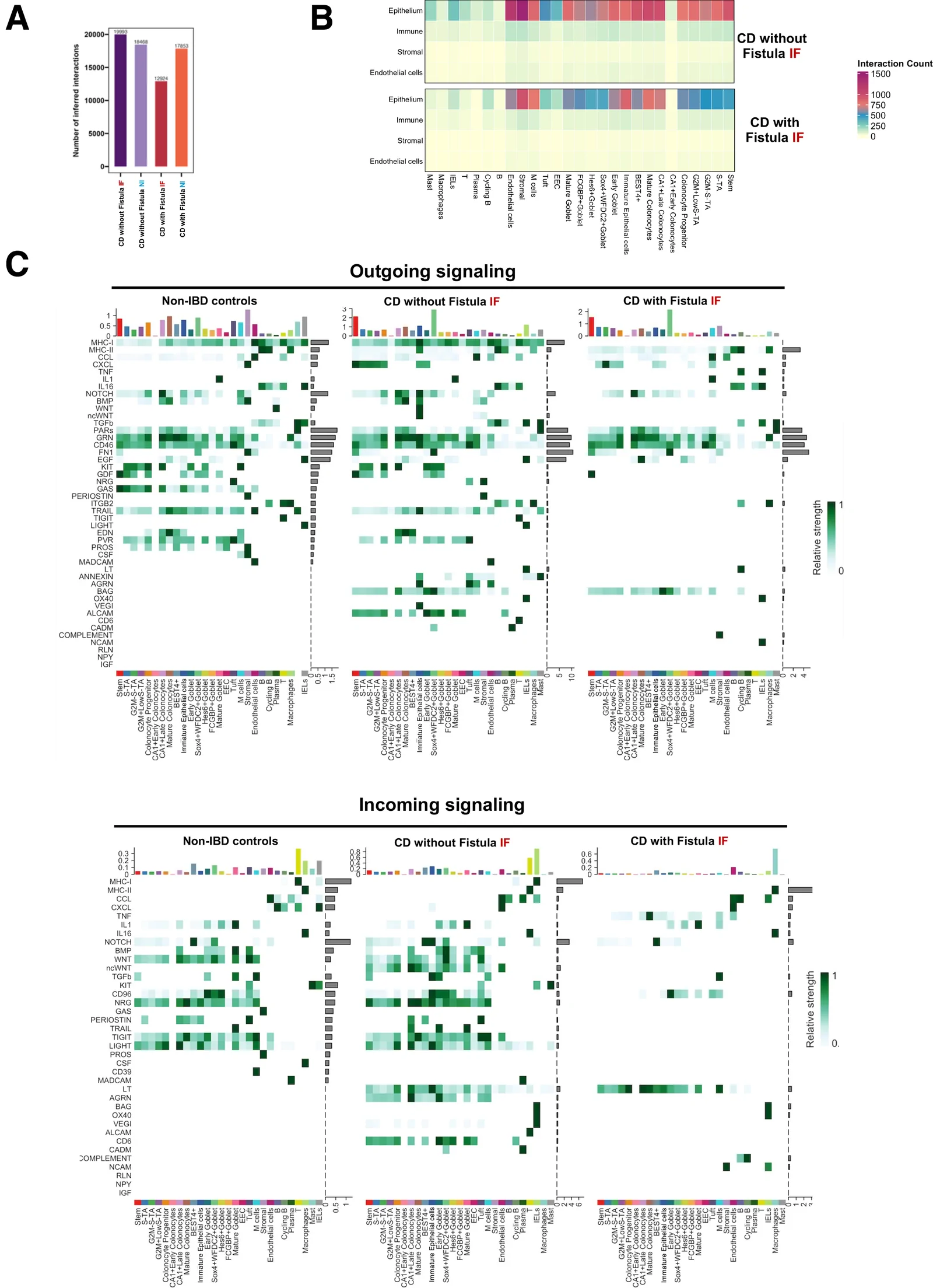
**Receptor–ligand interaction analysis of mucosal cells in IF CD of the rectum with and without the fistulizing phenotype.** (*A*) Bar graphs illustrating the cumulative number of receptor–ligand interactions in controls and CD of the rectum with and without fistulizing phenotype, with additional bar graphs highlighting the cumulative interactions stratified by the inflammatory phenotype. (*B*) Chord plots demonstrating inter-compartmental receptor–ligand interactions in IF CD of the rectum with and without fistula. (*C*) Heatmaps visualizing outgoing and incoming signaling patterns across different cell subtypes in controls and IF CD of the rectum with and without fistula.

**Figure 10:**
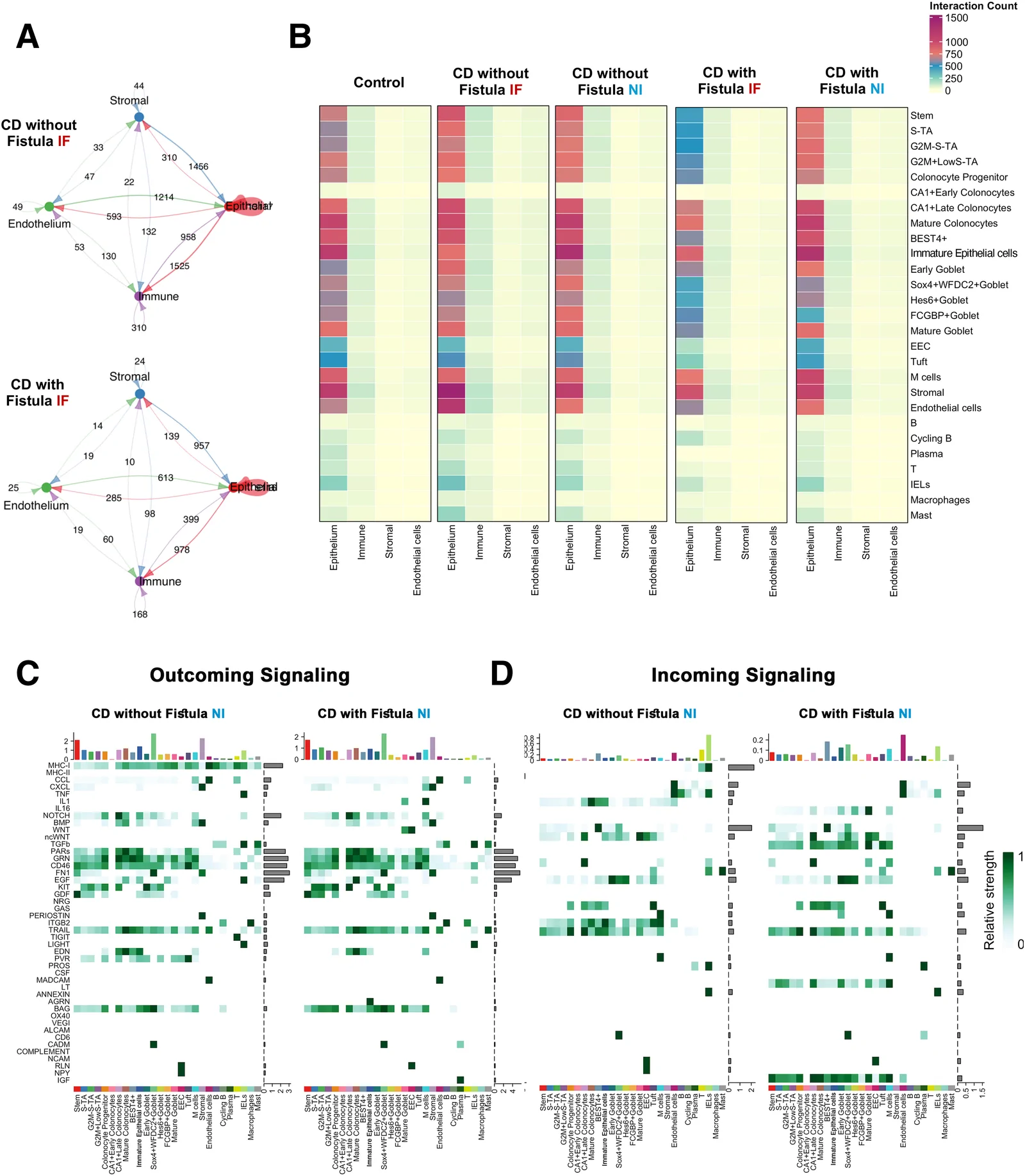
**Cell–cell communication stoichiometry highlights cellular distinctions.** (*A*) Chord plot showing the absolute number of receptor–ligand interactions between the rectal mucosal compartments. (*B*) Heatmap displaying the number of receptor–ligand interactions between individual cell type and mucosal compartments. (*C* and *D*) Heatmap illustrating relative signaling strength across the NI rectal phenotypes.

A detailed examination of the cellular networks showed that individuals with CD of the rectum and fistula, regardless of their inflammation, had no appreciable inferred signals for MHC class I antigen presentation pathways across all their mucosal cell types (Figure 9*C* and Figure 10). In addition, there was obvious loss of signals for WNT, bone morphogenic protein, TRAIL, PVR, and PROS for IF CD of the rectum with fistula, whereas activated leukocyte cell adhesion molecule signatures were unique to IF CD of the rectum without fistula. This was supported by diminished interactions between stromal and epithelial cells during fistulizing disease (Figure 10). There were also detectable differences in WNT and CCL2 expression levels among the stromal cells from different phenotypic groups (Figure 11). Only control cells indicated expression of *WNT2B* and *WNT5B* as compared with IF CD of the rectum with and without fistula. Unique to nonfistulizing CD in the stromal compartment was the increased expression of *CXCL1* and *WNT5A* (Figure 11). The signaling between epithelial cells was also diminished during mucosal inflammation in fistulizing disease, comparatively, as many of the epithelial–epithelial signals are intact in IF CD without fistula (Figure 10), implicating NOTCH in these processes. This is supported by a significant increase in NOTCH signaling during IF CD without fistula (Figure 5*C*). Interestingly, we also inferred differences in MHCII activity between IF CD of the rectum with and without fistula, whereby nonfistulizing disease primarily showed enrichment for antigen presentation to T cells, whereas fistulizing disease reflected a higher enrichment for antigen presentation to macrophages (Figure 9*C*).

**Figure 11:**
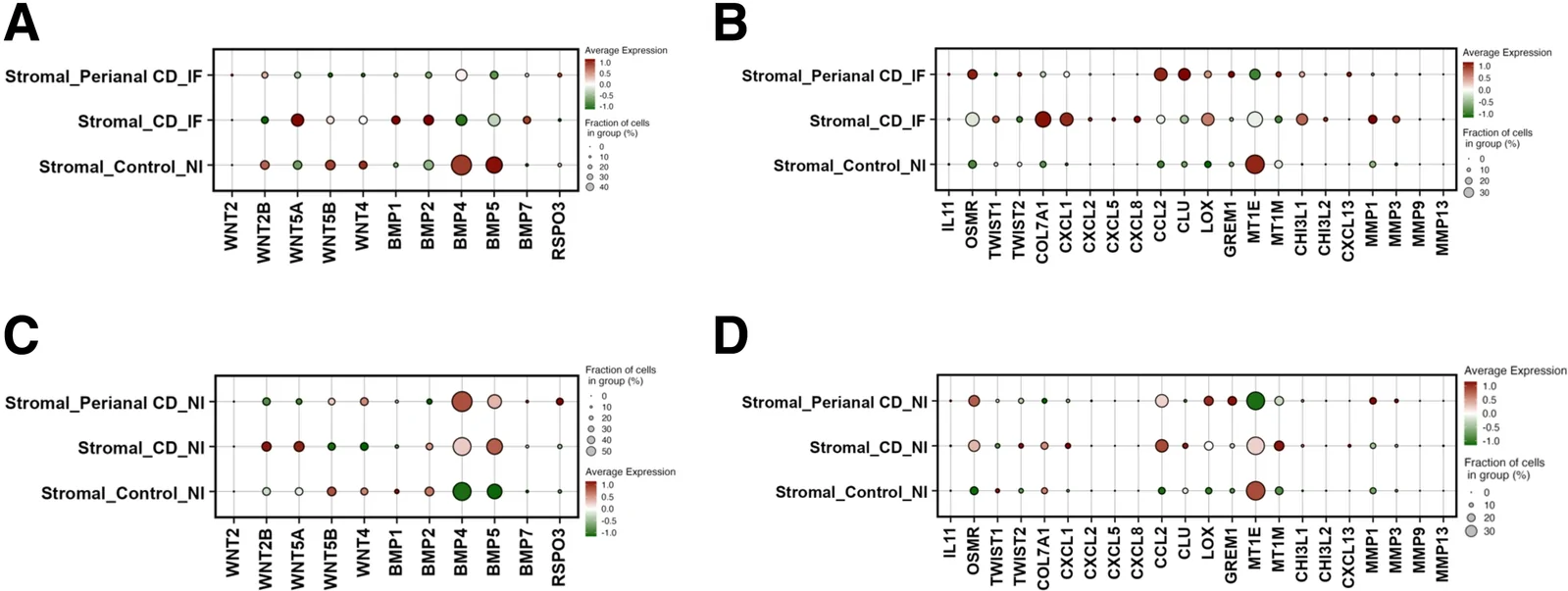
**Gene expression patterns in the stromal compartment.** (*A* and *B*) Dot plot highlighting the relative expression of WNT and bone morphogenic protein (BMP) pathway genes involved in epithelial proliferation and differentiation. (*C* and *D*) Dot plot showing expression of genes associated with inflammation-associated fibroblasts.

### Antigen Presentation to Macrophages in Inflamed Crohn’s Disease of the Rectum With Fistula

We further investigated differences in the antigen presentation/MHCII signaling in IF CD of the rectum with fistula (Figure 12*A*) and inferred from our single-cell data that macrophages were receiving epithelial MHCII signaling via the CD4 receptor (Figures 12 and 13). Closer examination of *CD4* expression showed that, in addition to T cells, ∼15% of the macrophages were found to be *CD3D*^−^*CD68*^+^*CD4*^+^ in their expression pattern (Figure 12*B*), prompting further investigation of this macrophage population.

**Figure 12:**
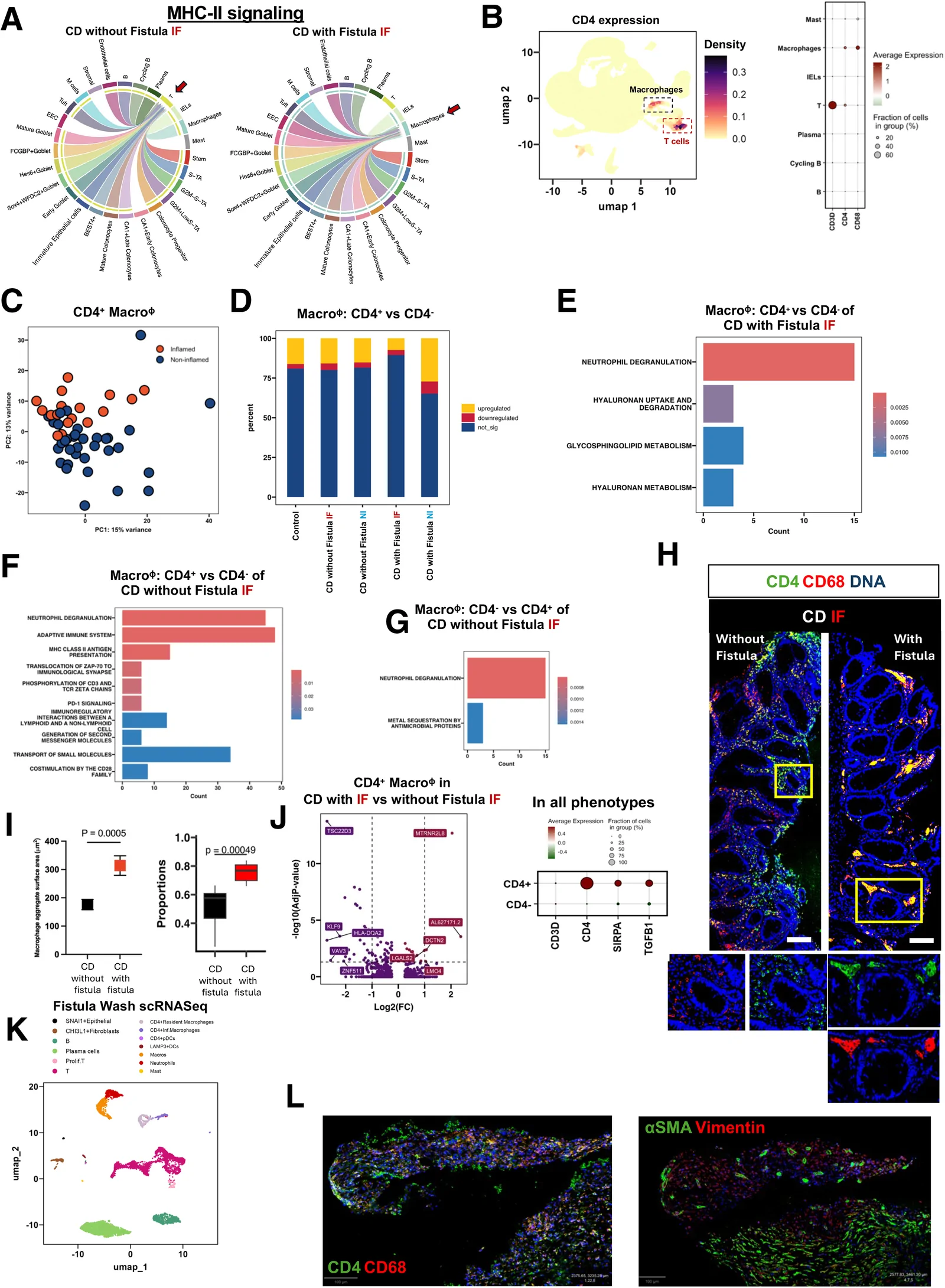
**Mucosal CD4^+^/CD68^+^ macrophages show unique morphology and characteristics in IF CD of the rectum with fistula.** (*A*) Chord plots showing results of receptor–ligand pair analysis, highlighting incoming MHCII signaling from epithelial subtypes to T cells in IF CD of rectum without fistula and towards macrophages in IF CD of the rectum with fistula. (*B*) UMAP of integrated scRNA-seq data highlighting *CD4* expression within the macrophage population, accompanied by a corresponding dot plot comparing *CD4*^*+*^ and *CD4*^*−*^ macrophages and their expression of common macrophage markers. (*C*) PCA showing that the IF CD phenotype accounts for 13% of the variance observed among *CD4*^*+*^ macrophages. (*D*) Differentially expressed genes of *CD4*^*+*^ macrophages compared with *CD4*^*−*^ macrophages, showing the greatest differences in NI CD of rectum with perianal complications and the least differences in IF CD of the rectum with fistula (*E*) Pathway analysis results showing significantly upregulated pathways in *CD4*^+^ macrophages from IF CD of the rectum with fistula compared with their *CD4*^−^ counterparts. (*F*) Pathway analysis results showing significantly upregulated pathways in *CD4*^*+*^ macrophages from inflamed CD of the rectum without fistula compared with their *CD4*^*−*^ counterparts. (*G*) Pathway analysis results showing significantly upregulated pathways in *CD4*^*−*^ macrophages from IF CD of the rectum with fistula compared with their *CD4*^*+*^ counterparts. (*H*) Immunofluorescence images of formalin-fixed paraffin-embedded biopsy tissue sections from IF CD of the rectum with and without fistula, showing colocalization of CD4^+^CD68^+^ macrophages in both phenotypes. (*I*) Box and whisker plots showing the measured surface area of macrophage aggregates quantified using QuPath and the relative cell abundance of CD4^+^ macrophages from tissue segmentation analysis of immunofluorescence images, highlighting a significantly higher mean surface area and increased CD4^+^ macrophage abundance in IF CD with perianal complications. (*J*) DEGs of *CD4*^*+*^ macrophages in IF CD of the rectum with and without fistula, highlighting upregulation of genes associated with M1-type macrophage polarization in the fistulizing phenotype. (*K*) UMAP of integrated RNA-seq data from perianal fistula tissue samples in CD of the rectum highlighting the presence of *CD4*^*+*^ macrophages within the fistula tract. (*L*) Immunofluorescence images of formalin-fixed, paraffin-embedded fistula tissue fragment showing colocalization of CD4^+^CD68^+^ macrophages (*left*) and presence of fibroblasts (*right*) in the fistula tract. α-SMA, α-smooth muscle actin.

**Figure 13:**
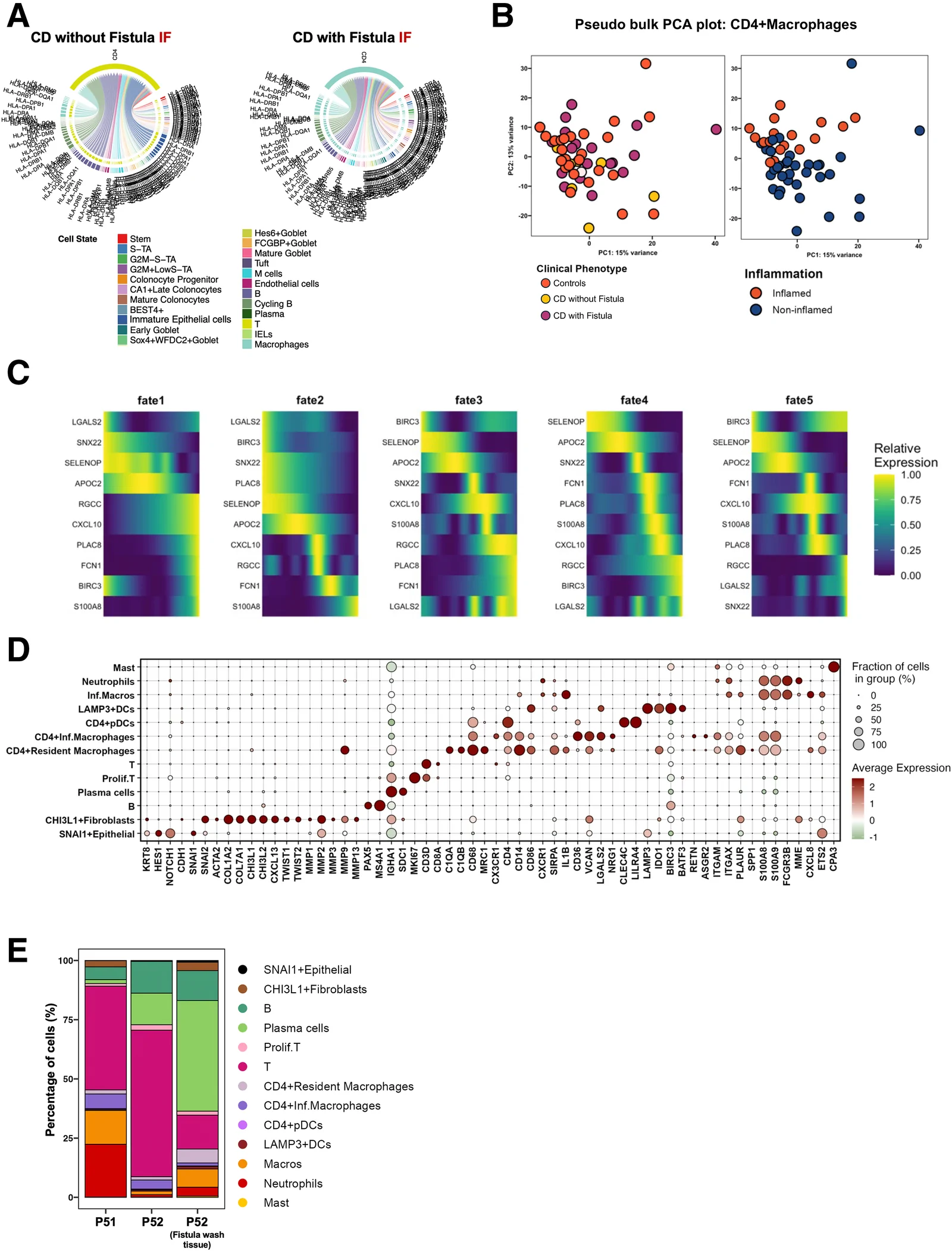
**Immune signaling and macrophage heterogeneity in CD.** (*A*) Chord plot depicting receptor–ligand interactions involved in the MHCII signaling in CD with and without fistula. (*B*) Pseudobulk PCA plot showing inflammation as the primary source of variance in *CD4*^*+*^ macrophages, with no separation by clinical phenotype. (*C*) Heatmap illustrates the relative expression of the top variable genes across the macrophage subcluster fates. (*D*) Dot plot of selected marker genes used for cluster annotation in fistula single-cell data. (*E*) Distribution of fistula-associated cell-type proportions across 3 fistula samples.

To further characterize the biological features of these CD4^+^ macrophages, we next examined their transcriptional profiles across disease phenotypes and inflammatory states. The IF status of the patients from whom biopsies were obtained accounted for the greatest degree of transcriptional variance among *CD4*^*+*^ macrophages (Figure 12*C* and Figure 13). The largest changes in transcriptional differences between macrophages with and without *CD4* expression were in noninflamed (NI) CD of the rectum with fistula, where *CD4*^+^macrophages showed many upregulated genes, and IF CD of the rectum with fistulizing phenotype showed the fewest (Figure 12*D*). Pathway analysis revealed that *CD4*^+^macrophages in IF CD of the rectum with fistula showed increases in hyaluronan uptake and metabolism and lipid metabolism pathways as compared with macrophages that were not expressing *CD4* (Figure 12*E*). This contrasted with *CD4*^+^ macrophages in IF CD of the rectum without fistula compared with their counterparts that were not expressing *CD4*, reflecting changes associated with MHCII, zeta-chain–associated protein kinase 70, PD-1, and CD28 pathways (Figure 12*F* and *G*).

To validate these transcriptional inferences, we stained mucosal biopsy sections from IF regions of CD of the rectum with fistula using antibodies for CD4 and CD68 and compared the results with similarly active CD of the rectum without fistula. This revealed clear morphologic distinctions in both the degree of colocalization of the CD4 and CD68 antibody signals and the larger aggregates uniquely in the lamina propria of the CD of the rectum with fistula (Figure 12*H* and *I*). This further establishes differences in the biology of these cells between the fistulizing and nonfistulizing phenotypes, as indicated by the single-cell data. Comparing transcriptomes of *CD4*^+^ macrophages between IF CD of the rectum with and without fistula showed changes in gene expression related to macrophage polarization and proinflammatory signaling.^22^ Interestingly, the *CD4*^+^ macrophage phenotype is further substantiated by their *SIRPA* expression (Figure 12*J*) that we recently found to be proinflammatory and associated with anti-TNF–refractory disease.^23^

We also sequenced cells harvested from perianal fistula washes from 3 samples and detected transcriptional evidence for the presence of several immune cell types, including *CD4*^+^ macrophages (Figure 12*K* and Figure 13*D* and *E*). Furthermore, intact tissue that was recovered in the fistula wash stained positively for CD4 and CD68, with some cells coexpressing both markers (Figure 12*L, left*), giving further evidence for the involvement of these cells in fistulizing disease. Interestingly, the same piece of fistula-associated mucosal tissue also stained positively for an unusually large number of cells expressing α-smooth muscle actin and vimentin (Figure 12*L, right*), suggesting a solid, fibrotic-like composition to the tissue associated within the fistula tract.

### Dynamics of *CD4*^*+*^ Macrophages in Inflamed Crohn’s Disease of the Rectum With Fistula

We further analyzed the macrophages in Figure 12*B* across the CD phenotypes by subsetting the macrophage cluster and determining cellular subtypes, proportions, and profiles of gene expression (Figure 14). The subclustering revealed more granular myeloid and phagocyte cell types, such as dendritic cells and neutrophils, apart from macrophage subtypes (Figure 14*A* and Figure 13). We identified 3 macrophage subpopulations: (1) resident macrophages without *CD4* expression; (2) resident macrophages with *CD4* expression; and (3) inflammatory macrophages that expressed *CD4*. Furthermore, we detected increased neutrophil proportions in nonfistulizing IF CD of the rectum, whereas higher proportions of *CD4*^+^ inflammatory macrophages were found in IF CD of the rectum with fistula (Figure 14*A–C*).

**Figure 14:**
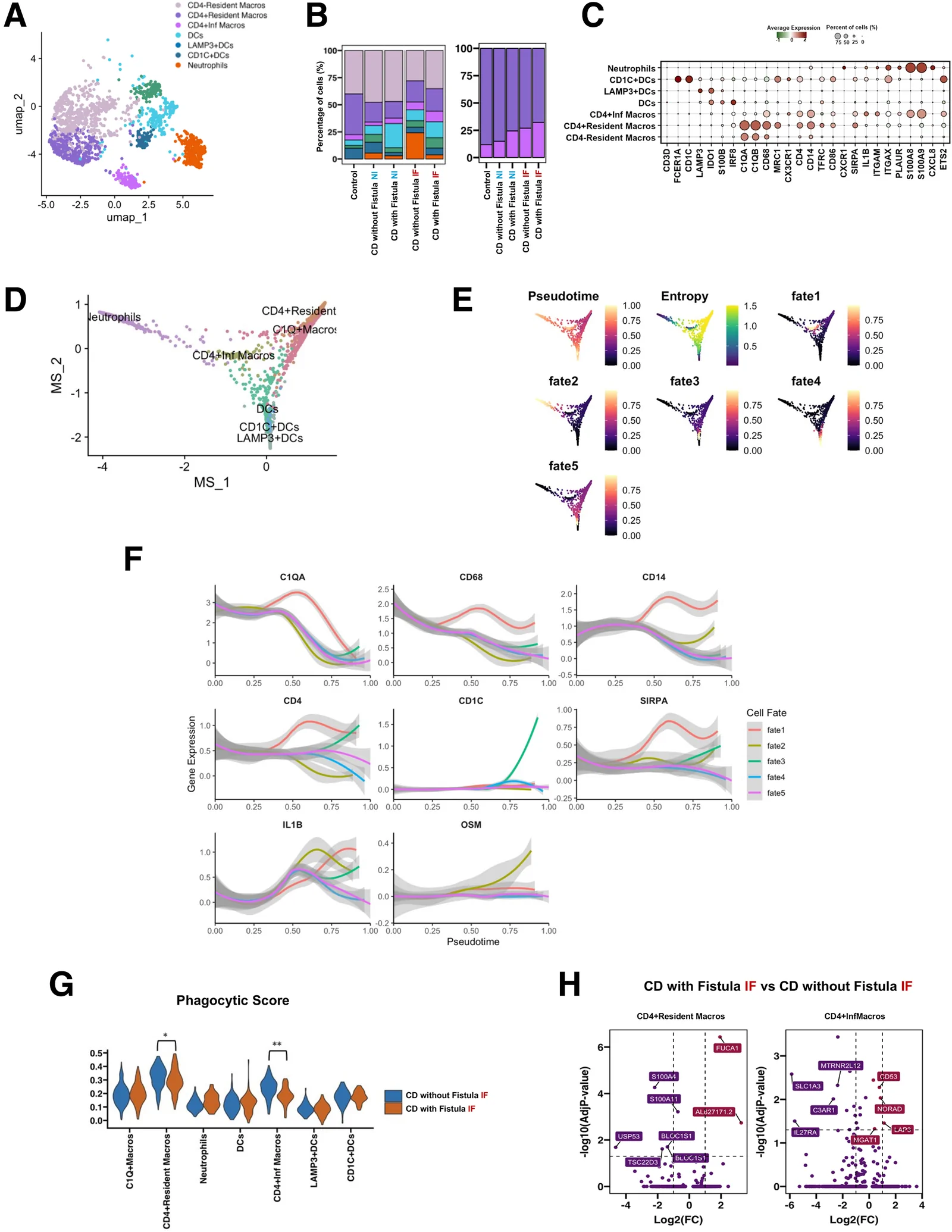
***CD4*^*+*^ macrophage subclusters demonstrate reduced phagocytic activity in IF CD of the rectum with perianal fistula.** (*A*) UMAP visualizing myeloid subtypes in integrated scRNA-seq data. (*B*) Corresponding bar plots showing the relative proportions of myeloid clusters across different phenotypes, along with a bar plot illustrating the relative proportions of *CD4*^*+*^ inflammatory and resident macrophages among phenotypes, highlighting an increased proportion of *CD4*^*+*^ inflammatory macrophages in IF CD with and without fistula compared with other groups. (*C*) Dot plot visualizing the expression of common macrophage marker genes across macrophage subclusters. (*D*) UMAP visualizing the pseudotime analysis results of 6 distinct myeloid clusters. (*E*) UMAPs highlighting the pseudotime, entropy, and different transcriptomic fates identified across the macrophage subtypes. CD4+ macrophage population lies in fate 1. (*F*) Plots showing the expression of selected genes of interest across different cellular fates, highlighting higher relative expression of *CD68*, *CD14*, *CD4*, *IL1B*, and *SIRPA* in fate 1. (*G*) Phagocytic module analysis of macrophage subclusters showing a significantly reduced phagocytic score in *CD4*^*+*^ resident and CD4^+^ inflammatory macrophages from CD with fistula compared with those from CD without fistula. (*H*) Comparative bioanalytics using single cell RNA-seq data of *CD4*^*+*^ resident macrophages (*left*), or of *CD4*^*+*^ inflammatory macrophages in perianal CD vs CD.

Pseudotime algorithms were used to examine the lineage progression of *CD4*^+^ macrophages with resident macrophages that were not expressing *CD4* as the starting timepoint. Five distinct cellular fates were detected, with the shortest interval being associated with the *CD4*^+^ inflammatory macrophages that emerged before the separation towards the neutrophil-like and dendritic cells (Figure 14*D* and *E*). The fate1 output for the *CD4*^+^ inflammatory macrophage lineage shows the associated transitionary gene expression profile (Figure 13*C* and Figure 14*E*). In comparison to all 5 of the calculated fates (Figure 13*C*), fate1, associated with *CD4*^+^ inflammatory macrophages, demonstrated consistent expression of *CD68*, *CD14*, *SIRPA*, and *CD4* through the time interval but had increased expression of *IL1B* and low levels of *CD1C* and *OSM* expression (Figure 14*F*), further establishing a proinflammatory phenotype. *CD4*^+^ macrophages from IF CD of the rectum with fistula also showed a reduced phagocytic score (Figure 14*G*). When compared with IF CD of the rectum without fistula, both resident and inflamed *CD4*^+^ macrophages in fistulizing disease show M2-type gene upregulation^24^ (Figure 14*H*).

### *CD4*^+^ Macrophages Colocalization With T Cells Signifying Perianal Fistulizing Crohn’s Disease

Next, we investigated the functional involvement of *CD4*^+^ macrophages within the spatial architecture of the rectal mucosa. We analyzed the mucosal microdomains present in inflamed CD of the rectum with and without fistula, with rich *CD4*^+^ macrophages by spatial transcriptomic analysis of 10 patients (6 patients with IF CD of the rectum without fistula and 4 patients with fistula; 210K cells and 322 gene probes) (Figure 15*A* and *B*). Because the colon gene panel used in this study did not have a *CD4* probe, we substantiated the presence of *CD4*^+^ macrophages in pan-gastrointestinal single-cell data and used this to anchor onto the segmented Xenium data.^25^ The *CD4*^+^ macrophage subtype in the lamina propria generated more granular cell types in Xenium compared with colon probes alone, and validated epithelial, immune, and stromal compartments in their designated spatial location (Figure 15*C*). Of the 5 macrophage subtypes from the pan-gastrointestinal atlas,^26^ macrophage_MMP9 and macrophage_CD5L showed *CD4* expression, which enabled us to detect and spatially impute the localization of *CD4*^+^ macrophages on the Xenium imagery platform (Figure 16). From here onward, we refer to them as *CD4*^*+*^*MMP9*^*+*^ *macrophage* and *CD4*^*+*^*CD5L*^*+*^ *macrophage*. *C1QA* expression *(*a macrophage marker from xenium probes) colocalizing *CD4*^+^ macrophages subtypes in the lamina propria additionally confirmed macrophage localization (Figure 15*D*). This method was further validated by localizing CD4^+^CD68^+^ macrophages in serial sections of the same tissue by immunofluorescence (Figure 15*E*). Across all the analyzed tissue**,** IF CD of the rectum with and without fistula showed similar cellular proportions for both mucosal compartments and *CD4*^+^ macrophage subtypes (Figure 17). Therefore, we conducted a neighborhood analysis to uncover more granular data from spatial tissue.

**Figure 15:**
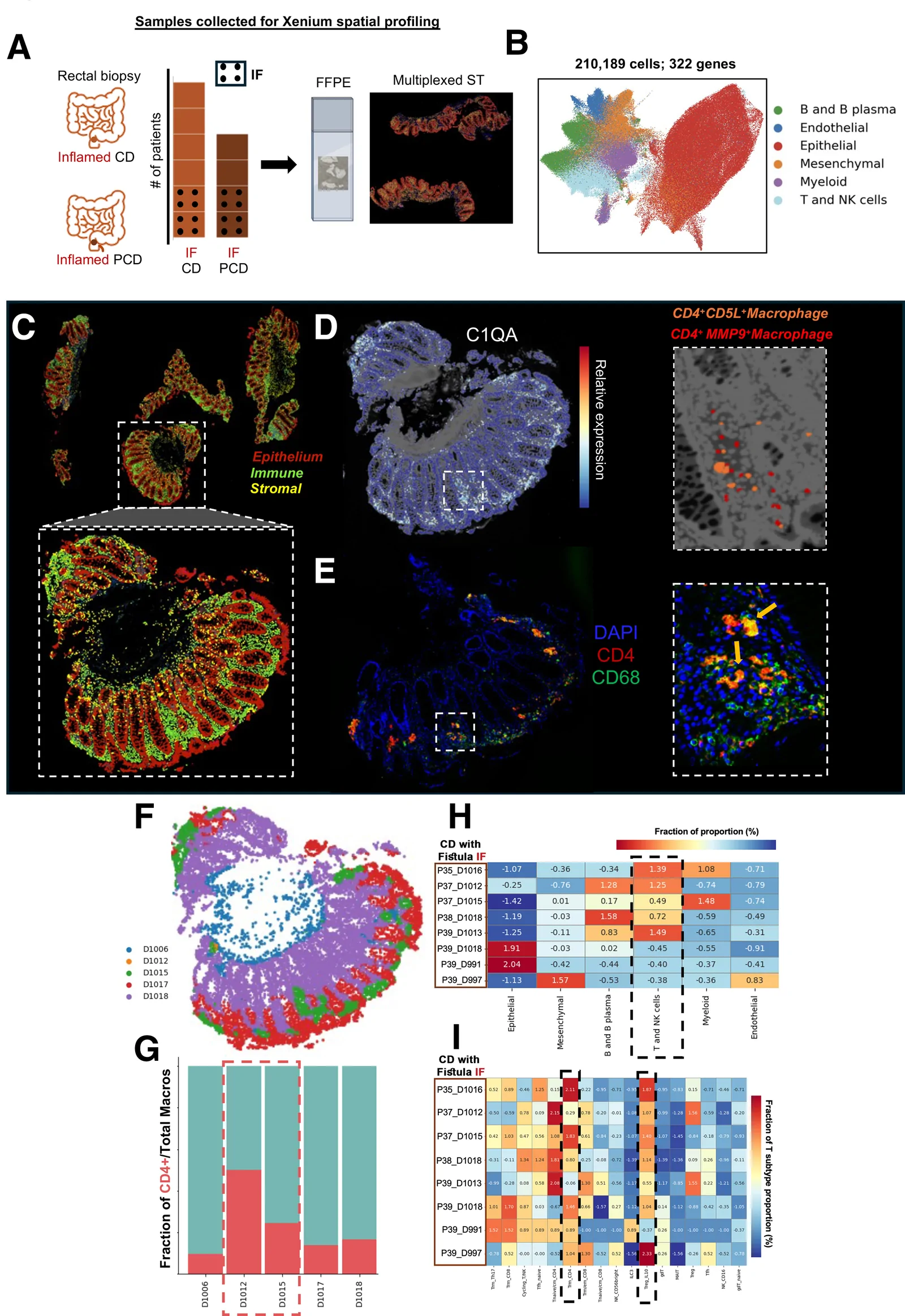
**Spatial transcriptomics of rectal biopsy sections reveal *CD4*^*+*^ macrophage colocalization with T cells in CD of the rectum with fistula.** (*A*) Graphical presentation of the arrangement of formalin-fixed paraffin-embedded (FFPE) tissue sections used in spatial transcriptomics (n = 10; 6 CD and 4 perianal CD [PCD]). (*B*) UMAP distribution of compartments. (*C*) Representative plot of spatial localization of epithelial, stromal, and immune compartments. (*D*) Colocalization of *C1QA* with *CD4*^*+*^ macrophages in spatial tissue. (*E*) Serial section of FFPE tissue highlighting the CD68^+^CD4^+^macrophages localizing to the same region in Figure 7*D*. (*F*) Five distinct neighborhoods were identified by spatial transcriptomics. (*G*) Representative bar plot of relative abundance on *CD4*^+^ macrophages in the neighborhoods. (*H*) Heatmap of *CD4*^*+*^ macrophage–high domains across patients with IF CD of the rectum with fistula, highlighting the colocalization of T and natural killer (NK) cells with *CD4*^+^ macrophages (n = 4). (*I*) Heatmap of T cell subtypes colocalization with *CD4*^+^ macrophages in microdomains of IF CD of the rectum with fistula.

**Figure 16:**
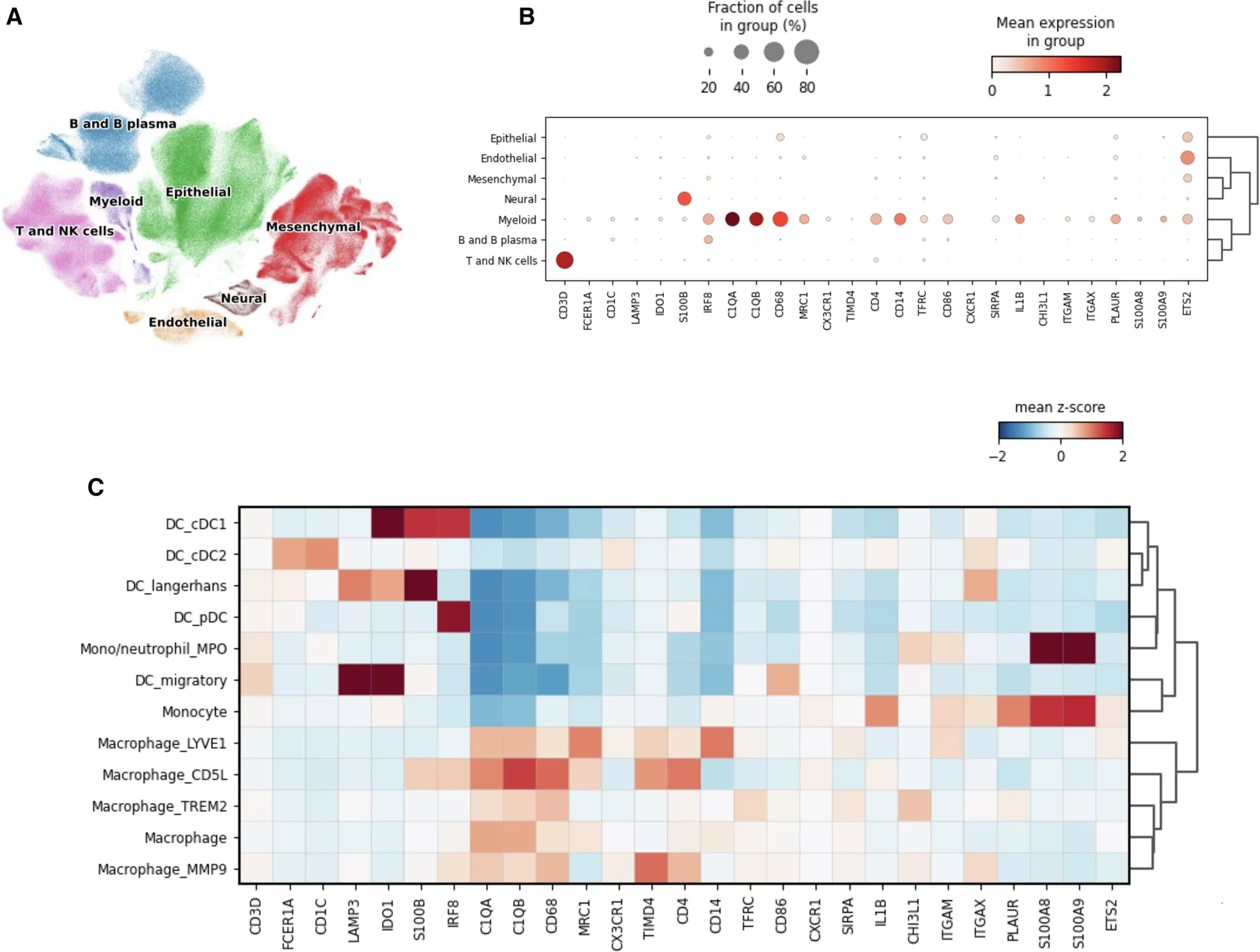
**Pan-gastrointestinal (GI) single-cell atlas data.** (*A*) UMAP showing major cell populations in the Pan-GI single-cell atlas used as a reference for Xenium annotation. (*B*) Dot plot confirming CD4 expression within the myeloid compartment. (*C*) Heatmap highlighting macrophage_MMP9 and macrophage_CD5L populations with the highest CD4 expression.

**Figure 17:**
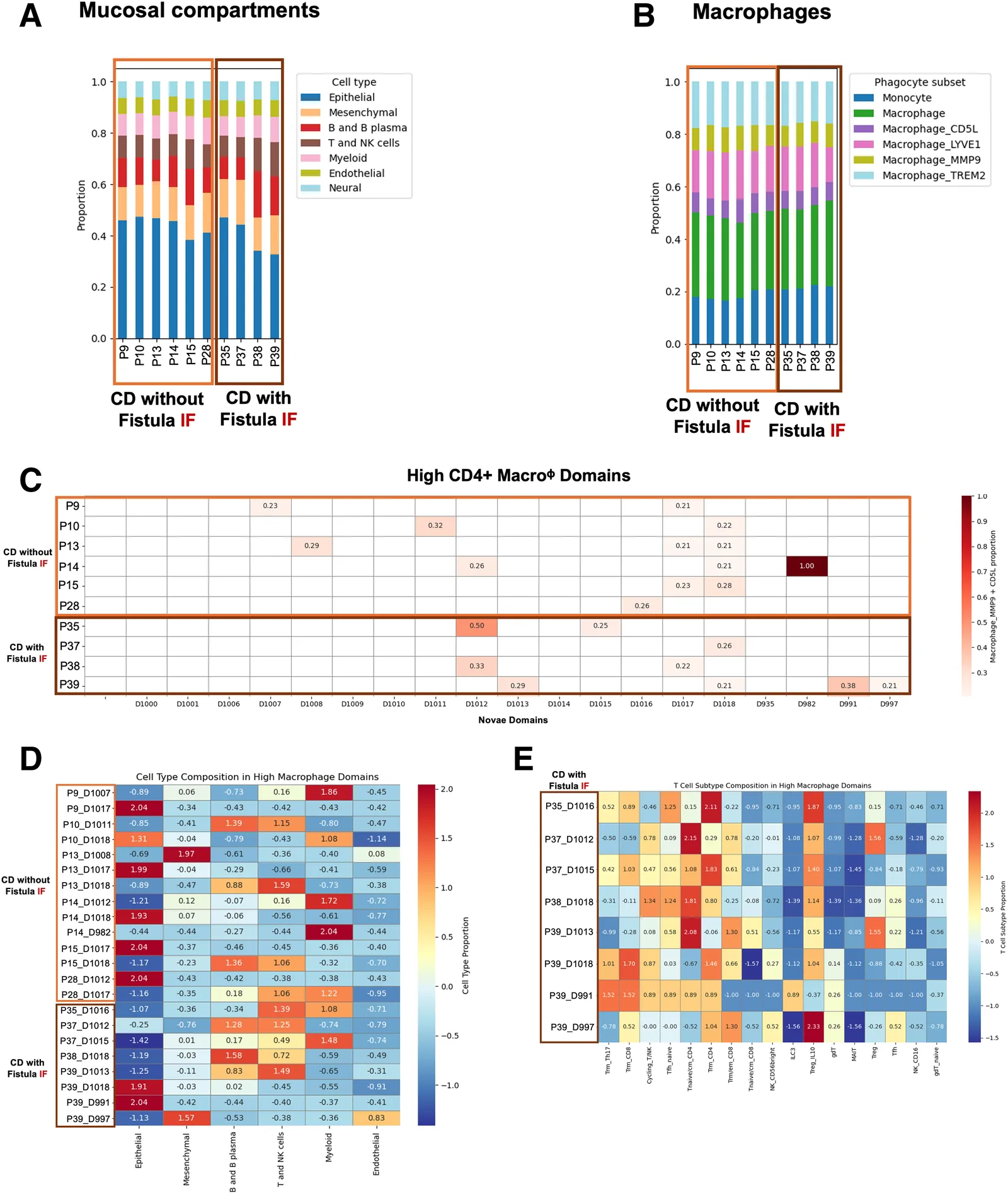
**Xenium spatial transcriptomic profiling of inflamed rectal mucosa.** (*A*) Stacked bar plot showing compartment distribution for each sample across CD phenotypes with and without fistula. (*B*) Stacked bar plot showing macrophage subtype distribution for each sample across CD phenotypes. (*C*) Heatmap highlighting spatial domains enriched for *CD4*^*+*^ macrophages (>20% of total macrophages). (*D*) Heatmap showing major compartment distribution within *CD4*^*+*^ macrophage–enriched domains. (*E*) Heatmap depicting T-cell subtype distribution within *CD4*^*+*^ macrophage–enriched domains.

To this end, bioanalytics of the tissue sections detected 5 broad neighborhoods within the mucosal sections in each tissue section (Figure 15*F*). Comparing the *CD4*^*+*^ macrophage proportion with total macrophages showed domains with relatively high *CD4*^*+*^ macrophages (>20%) (Figure 15*G* and Figure 17). Analyzing all the high *CD4*^*+*^ macro domains revealed that they are colocalized mostly with T and natural killer cells in IF CD of the rectum with fistula (Figure 15*H*). Further analysis on the T cell subtypes showed enriched Treg_IL10 and Trm_CD4 T cells in the *CD4*^*+hi*^ macrophage domains in fistulizing CD of the rectum, suggesting alternative cellular communication (Figure 15*H* and *I*).

## Discussion

The purpose of this study was to define molecular and cellular features that distinguish CD of the rectum with and without fistula, while accounting for ancestry as a biological variable. We increased the power of our previous study^12^ by recruiting additional patients (from 13 to 50) to enable a more in-depth analysis of rectal cells from EA and AA patients with scRNA-seq (+250K cells). There are only few comparative human population studies like this, making our results an important advancement in the mapping of cellular networks specific to individuals and populations associated with CD and the impact ancestry has on chronic inflammatory diseases.

A major finding of this study is the involvement of specialized proinflammatory CD4^+^ macrophages^27^ in fistulizing CD of the rectum. These cells were associated with increased T-cell activity in the rectal mucosa and appeared to affect epithelial cell function. The emergence, activation, and retention of this distinct CD4^+^ macrophage population may be driven by the loss of epithelial and stromal signaling cues observed in fistulizing CD, together with alterations in CCL2, that can further alter the chronic, dysregulated state of the macrophage compartment. Our findings also raise an important question on what causes the overwhelming CD4^+^ macrophage-specific MHCII antigen processing response in the lamina propria in IF CD of the rectum with fistula. Mechanistically, it could be the antigen presentation from epithelial cells that elicits the increased responses from CD4^+^ macrophages, or alternatively, the increased proportions of CD4^+^CD68^+^ macrophages in fistulizing CD of the rectum might be related to other types of cellular activity or homing mechanisms. Interestingly, these CD4^+^ macrophages express *SIRPA*, a macrophage subpopulation marker that was found to be enriched in the intestinal mucosa in anti-TNF–refractory CD.^23^ Thus, their presence in CD of the rectum with fistula may indicate a severe disease course that is characteristic of this phenotype.

Further analysis of these *CD4*^*+*^ macrophages in single-cell transcriptomics data revealed a M2 type polarization in IF CD of the rectum with fistula (Figure 14*H*). Consistent with this, our spatial transcriptomic analysis in IF CD of the rectum with fistula revealed that T cells colocalize with a CD4^+^ macrophage population, specifically resting memory CD4^+^ T cells and IL10-producing regulatory T cells (Tregs) (Figure 15*H* and *I*). IL10 derived from Tregs is an established signal promoting macrophage polarization toward an M2 phenotype,^28^ suggesting a potential mechanistic explanation for macrophage differentiation observed in our data. M2-polarized macrophages have been shown to enhance fibroblast activation and extracellular matrix synthesis,^29^ which might explain the increased abundance of fibroblasts observed in the tissue derived from the fistula tract (Figure 12*L*). Emerging evidence further supports the role of M2 macrophages in the progression of the fistula tract by their increased communication with fibroblasts.^21^ In addition, the MMP9 and CD5L in the M2 macrophages have previously been associated with tissue matrix remodeling and chronic inflammatory responses. Although M2 macrophages are traditionally considered anti-inflammatory, other lines of evidence suggest they can also play roles in the inflammatory process.^28^ Another notable observation from our study was the aggregation of macrophages in the IF rectum of fistulizing CD. Using functional assays, we substantiated the possibility that myeloid cell aggregation might be related to IL8-dependent signaling by the epithelium in a TNF-α–enriched milieu^23^^,^^30^ when we detected a significant increase in IL8 expression in rectal organoids after stimulation with TNF-α.

Given that 92% of patients with fistulizing disease exhibit CD of the rectum involvement,^31^ along with evidence suggesting that AA ancestry has a higher association with severe disease manifestation,^32^^,^^33^ we used 2 groups of individuals having either fistulizing perianal CD or having only CD with rectal involvement but without fistula or abscess. Collectively, the cohort was roughly equal in patients with EA and AA ancestry. At the population level and without regard to ancestry, some of the largest differences we observed were between IF CD of the rectum with and without fistula, indicating a major mucosal reprogramming occurring from the stages of IF CD of the rectum to the formation of fistula and abscess in these individuals. When considering ancestry, the IF CD of the rectum within the fistula group that were AA showed upregulation of many cellular pathways across multiple cell types, a pattern not observed in patients with EA ancestry. The increased cytokine and interleukin signaling across many cell types in AA patients with IF fistulizing disease pointed to an even higher level of immune activity in the rectum than observed in EA patients. These involved pathways were also the same features distinguishing IF CD of the rectum with fistula from nonfistulizing CD in AA patients, suggesting there are exacerbating immune triggers in the rectum during fistula development that are likely promoting an increase in pathways, such as IFN cascade, in AA patients. There are supporting genetic studies showing populations with African ancestry are associated with more robust immune responses,^34^ with up to 10% of macrophage-expressed genes linked to an African ancestral-specific response to inflammatory stimuli.^35^ Although our findings are consistent with a potential genetic contribution, other factors, including environmental, microbial, and epigenetic influences, are also taking part in these mechanisms and warrant future investigation.

At the phenotypic level, the most remarkable differences between IF CD of the rectum with and without fistula are taking place in pathways within epithelial cells that span the axis of the crypt. The overwhelming upregulation involving cytokine and IL signaling of the adaptive immune response and an increase in MHCII antigen presentation by epithelial cells, with activation of PD-1 and TCR signals occurring in IF CD of the rectum with fistula, shows that the rectal epithelium continues to undergo numerous functional changes in the progression of the disease. It is not clear, however, whether some of the epithelial cell metabolic pathway differences we observed between IF CD of the rectum with and without fistula reflect insults contributing to changes in epithelial barrier function caused by inflammation or are the initial drivers that facilitate and exacerbate the environment to form a fistula. We previously detected metabolic pathway changes in rectal organoids from patients with fistulizing CD,^13^ suggesting these changes are inherent to those individuals’ epithelial cells because no inflammatory mediators were used to stimulate organoids in those experiments. Furthermore, the changes in fatty-acid pathways in IF CD of the rectum with fistula link metabolic pathways to microbial activity in the pathology associated with increased MHCII complexes made by rectal epithelial cells during active disease. The association of abscess formation with this pathology and enrichment of the complement pathway in CD rectum with fistula also supports a potential microbial connection as another variable in determining disease progression. However, we did not consider microbial variables in this study. The sample size, limited number of gene probes for spatial experiments, and functional assays evaluating phagocytosis and antigen presentation in macrophage–organoid cocultures were limitations of our study. Nonetheless, we attempted to overcome this with multiple orthogonal approaches to strengthen and further substantiate our findings.

We provide a valuable data set of single-cell and spatial transcriptomics from multiple ancestral populations with CD of the rectum with and without fistula to be used for ongoing studies. Our experiments with rectal organoids indicate that the signals received by the epithelium during fistulizing disease promote high levels of STAT1, STAT6, and MHCII responses that need further investigation to better understand the connection between epithelial cells, T cells, and CD4^+^CD68^+^ macrophages during or just prior to the onset of fistula development. Future investigations should focus on the factors stimulating IFN pathways during CD of the rectum and their impact on specific subtypes of epithelial cells and macrophages using ancestral populations.

## Materials and Methods

### Patient Recruitment and Sample Collection

Mucosal biopsy samples were obtained from the rectum of patients with CD with and without perianal fistulizing disease during endoscopy procedures under an approved Emory University Institutional Review Board 2025P010865 protocol. Control biopsies were collected from non-IBD individuals with no evidence of intestinal inflammation during routine colonoscopy. These individuals underwent colonoscopy for abdominal pain or suspected colonic polyps and later were found to have normal mucosal macroscopically and microscopically. The CD cohort eligible for inclusion in this study represented an equal distribution of AA and EA ancestry. Patient metadata are summarized in Figure 2. The presence of mucosal inflammation was determined based on macroscopic (colonoscopy) assessment. A total of 50 individuals were enrolled in the study, including 25 with CD of the rectum without perianal disease (10 IF and 15 NI rectal mucosa) and 18 with CD of the rectum with perianal fistula (7 IF and 11 NI rectal mucosa). The remaining participants were non-IBD controls. Power calculations indicated that, with 80% power at *P* < .05, comparison of inflamed rectal mucosa from patients with CD without perianal disease (n = 10) vs those with perianal fistula (n = 7) would be sufficient to detect a mean difference of 1.48 standard deviations between groups. Three fistula wash samples (obtained during examination under anesthesia with surgeon), 1 with tissue fragments, were additionally collected from 2 individuals with perianal CD during seton placement.

### Single-Cell Isolation, Library Preparation, and Sequencing

Mucosal biopsies were protease-digested and processed for single-cell sequencing within 2 hours of endoscopy as previously described.^36^

### Single-Cell Data Processing

After sequencing, raw BCL files were demultiplexed using 10X Genomics Cell Ranger (v7.0.1), and the resulting FASTQ files were aligned to the human reference genome GRCh38 to generate feature–barcode matrices. Single-cell data processing and QC were performed using Seurat (v5.0.0).^37^ Cells expressing fewer than 250 or more than 7000 genes, as well as those with >50% mitochondrial content, were excluded. DoubletFinder^38^ was used to remove potential doublets. Mitochondrial and ribosomal genes were pruned in the QC. Count matrices from 50 samples were analyzed in Seurat. Unique molecular identifier counts were log-normalized, and highly variable genes were selected. Each sample was scaled prior to principal component analysis (PCA). To correct for batch effects, reciprocal PCA was applied, followed by dimensional reduction using PCA and projection via Uniform Manifold Approximation and Projection (UMAP) with 30 dimensions. Neighborhood analysis and clustering were performed using 20 dimensions at a resolution of 0.5, identifying broad cell populations.

### Cell Type Annotation and Subclustering

Clusters were annotated based on differentially expressed genes identified with the FindAllMarkers function, as well as known marker genes from published literature and cell-cycle phase information. Macrophage clusters characterized by *CD68*, *C1QA*, and *C1QB* expression were extracted from the integrated Seurat object. This macrophage subset was then normalized with SCTransform and integrated using the RunHarmony function.^39^ Subsequently, neighbor detection and clustering were repeated, and the data were visualized using UMAP at a resolution of 0.5. The resulting macrophage subclusters were annotated as in the opening section with published marker genes.^40^^,^^41^

### Inflammation, Crypt-–Axis, and Phagocytic Activity Scoring

The aggregate expression of signaling programs within the rectal mucosal scRNA-seq data was computed using the AddModuleScore function in Seurat. Gene sets to assess inflammation, crypt axis, and phagocytic programs were performed as previously described.^21^^,^^42^^,^^43^

### Cell–Cell Communication Analysis

CellChat^44^ with CellChatDB was employed to infer receptor–ligand interactions from the integrated rectal mucosal scRNA-seq dataset. Global signaling patterns were examined across cell populations within each phenotypic group, followed by pathway and ligand–receptor analysis.

### Pseudobulk Principal Component Analysis

Pseudobulk single-cell PCA plots were generated by aggregating raw unique molecular identifier count matrices of each sample to assess the transcriptomic variance.

### Differential Expressions and Pathway Analysis

Differential expression analysis was performed using the FindMarkers function and model-based analysis of single-cell transcriptomics. It was computed on cell types with at least 10 cells for each condition.

### Trajectory Analysis

To investigate phagocyte subpopulation dynamics, we performed pseudotime trajectory analysis using Palantir^45^ with default parameters, designating C1Q^+^ macrophages as the root cell population. Pseudotime estimation was used to infer directional cell-state transitions. In addition, fate probabilities were computed for each subpopulation, and the top fate-determining genes were identified and visualized using the CellRank package.

### Xenium Spatial Transcriptomics Protocol and Analysis

Regions exemplifying active and inactive sites of CD of the rectum with and without fistula within formalin-fixed paraffin-embedded tissue sections were identified by a board-certified pathologist. Selected slides were processed with Xenium 10X Genomics technology KIT#1000460, and human colon gene expression panel kit (1,000,642) on a Xenium Analyzer according to the manufacturer’s instructions. Xenium cell segmentation was carried out using a transcript-based Baysor algorithm. Analysis of probe signals/quality was performed with Xenium Explorer. Subsequent QC analysis and annotation were performed with Sopa.^25^ We assessed mucosal biology using neighborhood analysis with Novae^46^ and Squidpy,^47^ along with STLearn^48^ for receptor–ligand interactions.

### Immunofluorescence Microscopy and Quantification

Serial sections used for Xenium were also processed by standard histologic techniques. Briefly, after deparaffinization and heat-induced antigen retrieval using Diva Decloaker (BioCare Medical), slides were blocked in bovine serum albumin and stained with primary antibody (1:400 dilution; EPR6855 rabbit anti-CD4 from Abcam, mouse anti-CD68 from Abcam) overnight at 4 °C. Secondary antibodies (1:1000; Alexafluor from Thermo) and 4′,6-diamidino-2-phenylindole (1:2000) were applied for 1 hour at room temperature with multiple washes between steps. Background quenching was performed using Vector TrueVIEW Autofluorescence Quenching Kit according to the manufacturer’s protocol and mounted with Diamond Antifade mountant. Images were acquired using a PhenoImager HT 2.0 whole-slide scanner system, and downstream analysis on CD4^+^CD68^+^ signals were performed with QuPath software.

### Rectal Organoids, Western Blotting, and Quantitative Real-Time Polymerase Chain Reaction

Organoids were established from rectal biopsies as previously described.^49^ Organoids were maintained in a modified WNT3A, R-spondin, Noggin-based media, and stimulated with IFN-γ (1 ng/mL), IL13 (10 ng/mL), and TNF-α (25 ng/mL). Western blotting was performed essentially as described in,^49^ and the antibodies used were against the following: STAT1 Y701, STAT1 S727, STAT6 Y641, STAT1, STAT6, MHCII, and CD74. All these antibodies were obtained from Cell Signaling Technologies. Total RNA was extracted using the Qiagen RNeasy Mini Kit. Complementary DNA synthesis was performed using the Applied Biosystems TaqMan MicroRNA Reverse Transcription Kit. Quantitative real-time polymerase chain reaction was carried out using Fast TaqMan Master Mix, with CXCL8-specific primers obtained from Applied Biosystems.

### Statistical Analysis

Statistical analyses were performed to assess significant differences between phenotypic groups. The Wilcoxson test was used to compare cell abundance proportions, inflammation scores, phagocytic scores, and cell abundance proportions, whereas the simple *t* test was used to assess macrophage aggregate surface area within IF rectum tissue from patients with CD with and without fistula. REACTOME pathway enrichment analysis was conducted using significant differentially expressed genes (adjusted *P* value <.05 and log2fold change ≥1). Pathways with a false discovery rate–adjusted *P* value < .05 were considered significant.

## CRediT Authorship Contributions

Sushma C. Maddipatla, MS (Formal analysis: Lead; Methodology: Lead; Writing – original draft: Lead; Writing – review & editing: Lead)

Sachith Munasinghe, MD (Formal analysis: Lead; Investigation: Lead; Methodology: Lead; Writing – original draft: Lead; Writing – review & editing: Lead)

Anne Dodd, BS (Data curation: Equal; Investigation: Supporting; Methodology: Equal; Project administration: Lead; Resources: Lead)

Christopher Tastad, MS (Data curation: Lead; Methodology: Lead; Software: Equal)

Rachel Moss, MD (Formal analysis: Supporting; Methodology: Supporting; Software: Supporting)

Hong Yin, MD (Formal analysis: Supporting; Investigation: Supporting; Methodology: Supporting)

Shanta Murthy, MS (Data curation: Supporting; Formal analysis: Supporting; Methodology: Supporting; Software: Supporting)

Abhishek Dash, MS (Formal analysis: Supporting; Methodology: Supporting; Software: Supporting)

Vasantha L. Kolachala, PhD (Data curation: Supporting; Formal analysis: Supporting; Investigation: Equal; Methodology: Equal; Project administration: Lead; Resources: Lead; Writing – review & editing: Supporting)

David J. Cutler, PhD (Formal analysis: Supporting; Investigation: Supporting; Methodology: Supporting; Supervision: Supporting)

Judy Cho, MD (Data curation: Supporting; Investigation: Supporting; Methodology: Supporting; Resources: Equal; Supervision: Supporting; Writing – review & editing: Supporting)

Subra Kugathasan, MD (Conceptualization: Lead; Formal analysis: Supporting; Funding acquisition: Lead; Investigation: Supporting; Methodology: Supporting; Project administration: Supporting; Resources: Lead; Supervision: Equal; Writing – review & editing: Equal)

Jason D. Matthews, PhD (Conceptualization: Lead; Formal analysis: Lead; Funding acquisition: Supporting; Investigation: Equal; Methodology: Equal; Supervision: Lead; Writing – original draft: Lead; Writing – review & editing: Lead)
